# Supplementary material for: Assembly of a parts list of the human mitotic cell cycle machinery
Source: J Mol Cell Biol. 2018 Nov 17;11(8):703–18. doi: 10.1093/jmcb/mjy063 (PMC6788831; doi:10.1093/jmcb/mjy063)
Supplement: mjy063_Supplementary_Data_S1 [file mjy063_supplementary_data_s1.pdf]

Giotti et al., Supplementary Data 1. Results from subcellular localisation studies of uncharacterised proteins

For all images: HEK293T nuclei were stained with DAPI (blue), uncharacterised proteins were fused with GFP (green) and actin was stained with phalloidin and Texas Red (red). Scale bars = 10 µm.
Gene Symbol: TACC3

Gene description: transforming acidic coiled-coil containing protein 3
TACC3-GFP: Interphase: diffuse cytoplasmic signal (Figure 1A). Mitotic metaphase: signal at spindle poles (Figure 1B).
GFP-TACC3: Interphase: diffuse cytoplasmic signal (Figure 1C). Mitotic prophase: Punctate and fibrillar signal proximal to chromatin, diffuse signal elsewhere in cell (Figure 1D).
-N vs C- GFP expression: Consistent.

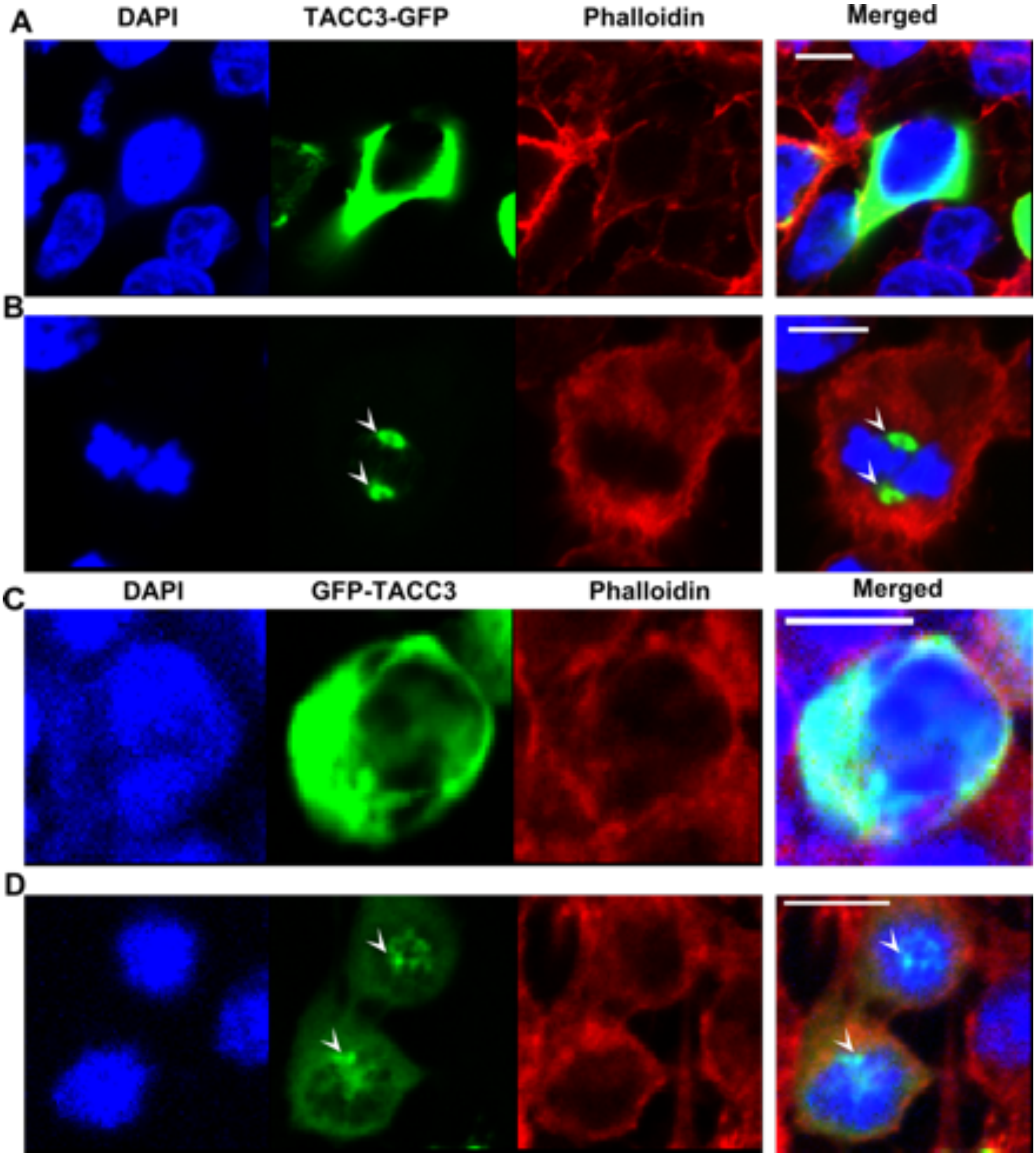

Figure 1. TACC3-GFP and GFP-TACC3 expression. (A) : Interphase: diffuse cytoplasmic signal. (B) Mitotic metaphase: signal at spindle poles. (C) Interphase: diffuse cytoplasmic signal. (D) Mitotic prophase: Punctate and fibrillar signal proximal to chromatin.

Gene symbol: CENPA

Gene description: centromere protein A
CENPA-GFP: Interphase: GFP signal is concentrated in nuclear puncta, aggregates and nucleoli. (Figure 2A). Mitotic prometaphase: Co-localisation with DNA (Figure 2B).
GFP-CENPA: Interphase: GFP signal is concentrated in nuclear aggregates (Figure 2C). Mitotic prometaphase: GFP signal is in chromatin foci (Figure 2D).
-N vs C- GFP expression: Consistent.

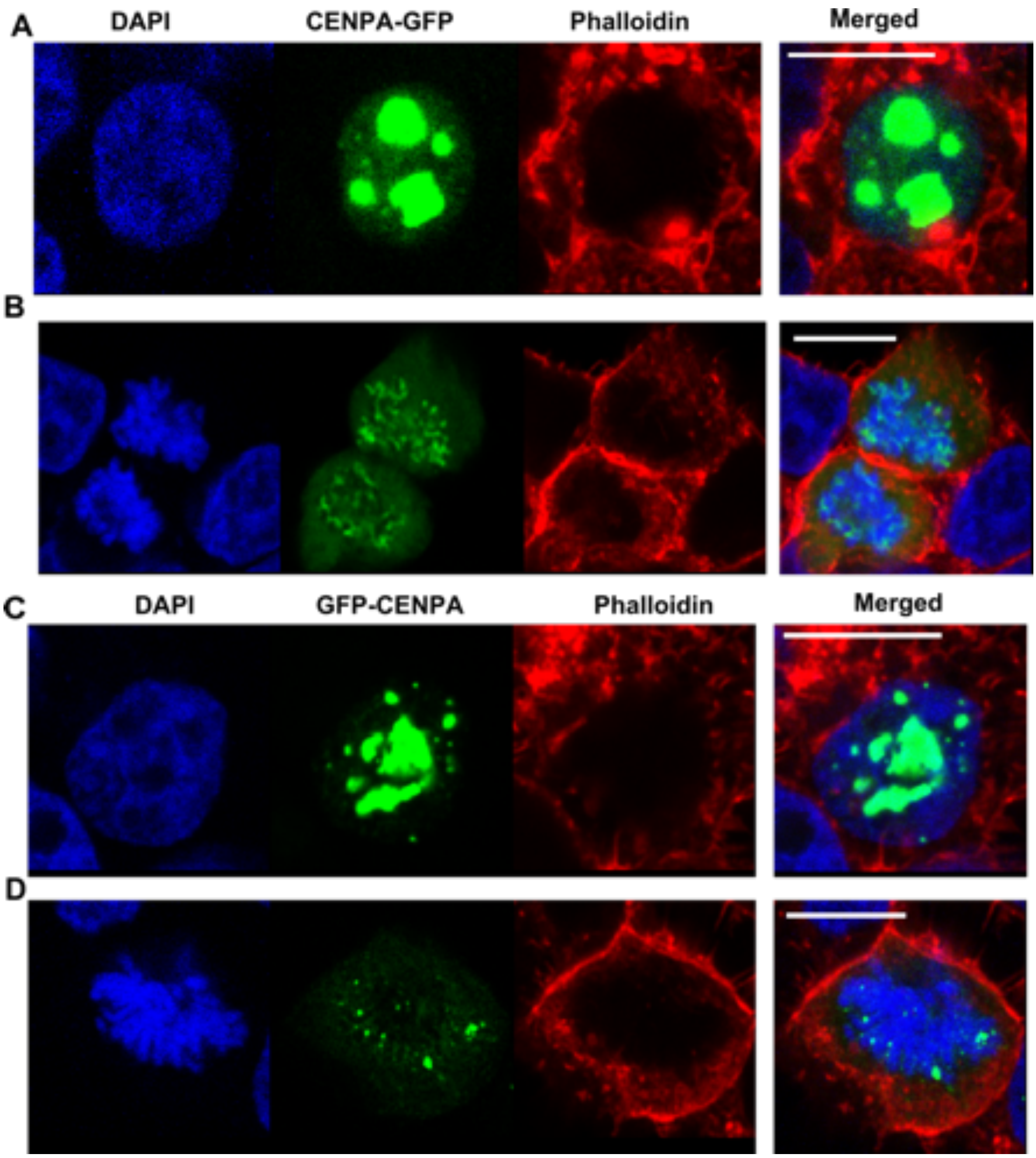

Figure 2. CENPA-GFP and GFP-CENPA expression. (A) Interphase: GFP signal is concentrated in nuclear puncta, aggregates and nucleoli. (B) Mitotic prometaphase: Co-localisation with DNA. (C) Interphase: GFP signal is concentrated in nuclear aggregates. (D) Mitotic prometaphase: GFP signal is in chromatin foci.

Gene Symbol*: C3orf14*

Gene description*:* chromosome 3 open reading frame 14
C3orf14-GFP*:* Interphase: GFP signal is concentrated in a single perinuclear focus (Figure 3A).
GFP-C3orf14*:* Interphase: GFP signal is concentrated in a single perinuclear focus. Mitotic prophase: GFP signal is concentrated in a single focus (Figure 4A). Mitotic metaphase: GFP signal is concentrated in three foci including two foci proximal to spindle poles (Figure 4B). Mitotic prometaphase: GFP signal is focused in two pair of dots proximal to condensed DNA (Figure 4C).
-N vs C- GFP expression: Consistent.

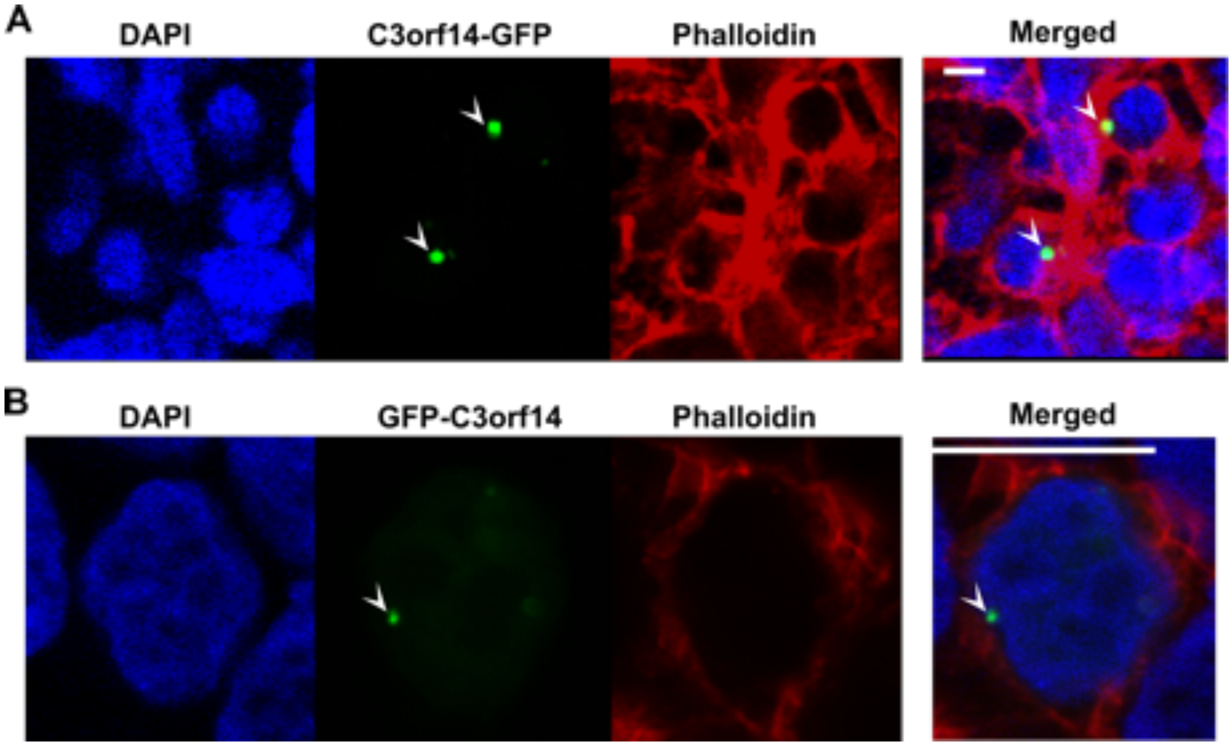

Figure 3. C3orf14-GFP and GFP-C3orf14 expression. Interphase: GFP signal is concentrated in a single perinuclear focus. (B) Interphase: GFP signal is concentrated in a single perinuclear focus.

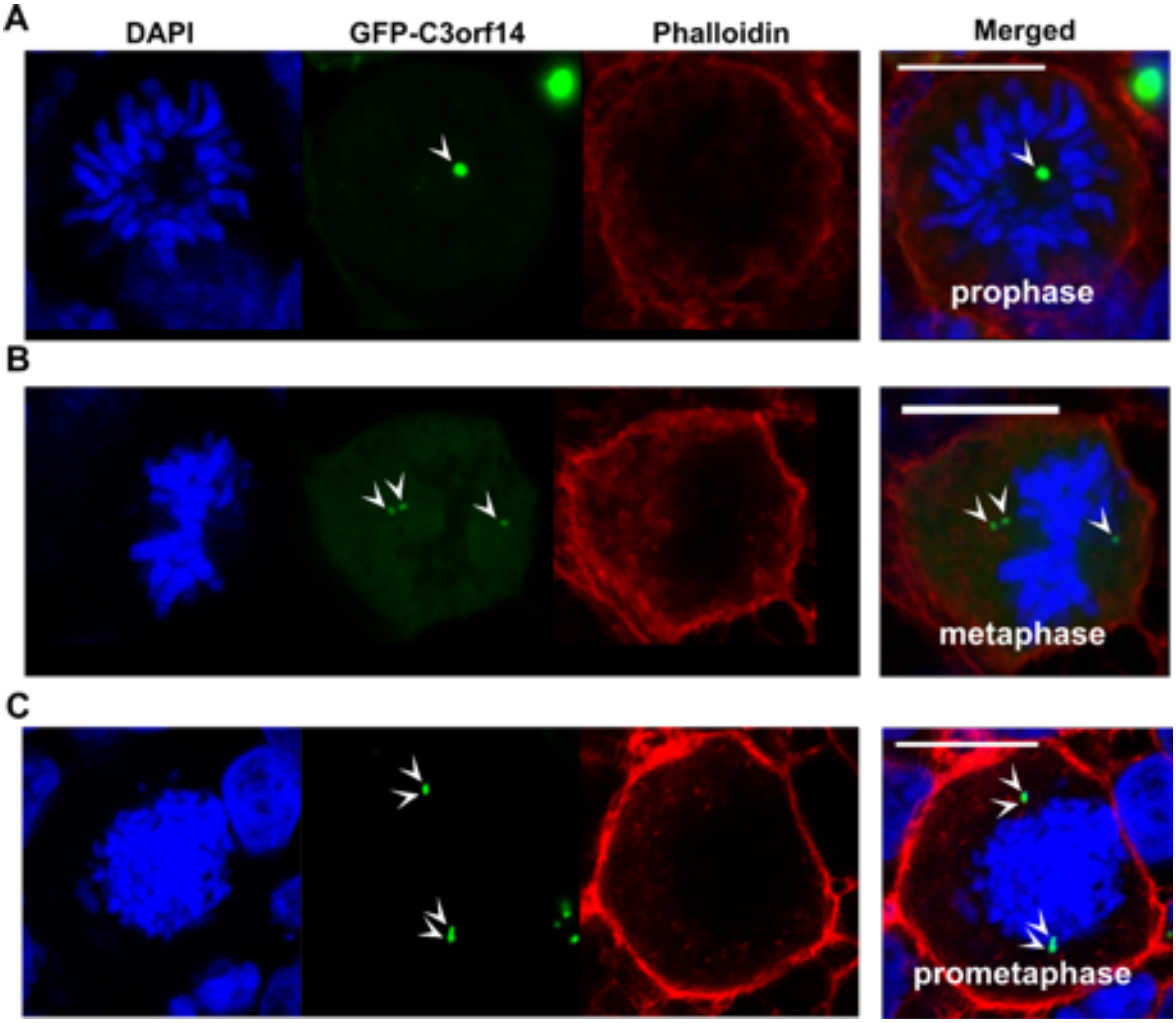

Figure 4. GFP-C3orf14 expression. (A) Mitotic prophase: GFP signal is concentrated in a single focus (arrow). (B) Mitotic metaphase: GFP signal is concentrated in three foci including two foci proximal to spindle poles (arrows). (C) Mitotic prometaphase: GFP signal is focused in two pair of dots proximal to condensed DNA (arrows).

Gene Symbol: ***CCDC150***

Gene description: coiled-coil domain containing 150

CCDC150-GFP: Interphase: GFP signal concentrated in perinuclear foci. (Figure 5A, arrows).

GFP-CCDC150: Interphase: GFP signal concentrated in perinuclear foci (Figure 5B, arrows). Mitotic prophase: GFP signal in punctate foci. (Figure 5C, arrows).

-N vs C- GFP expression: Consistent.

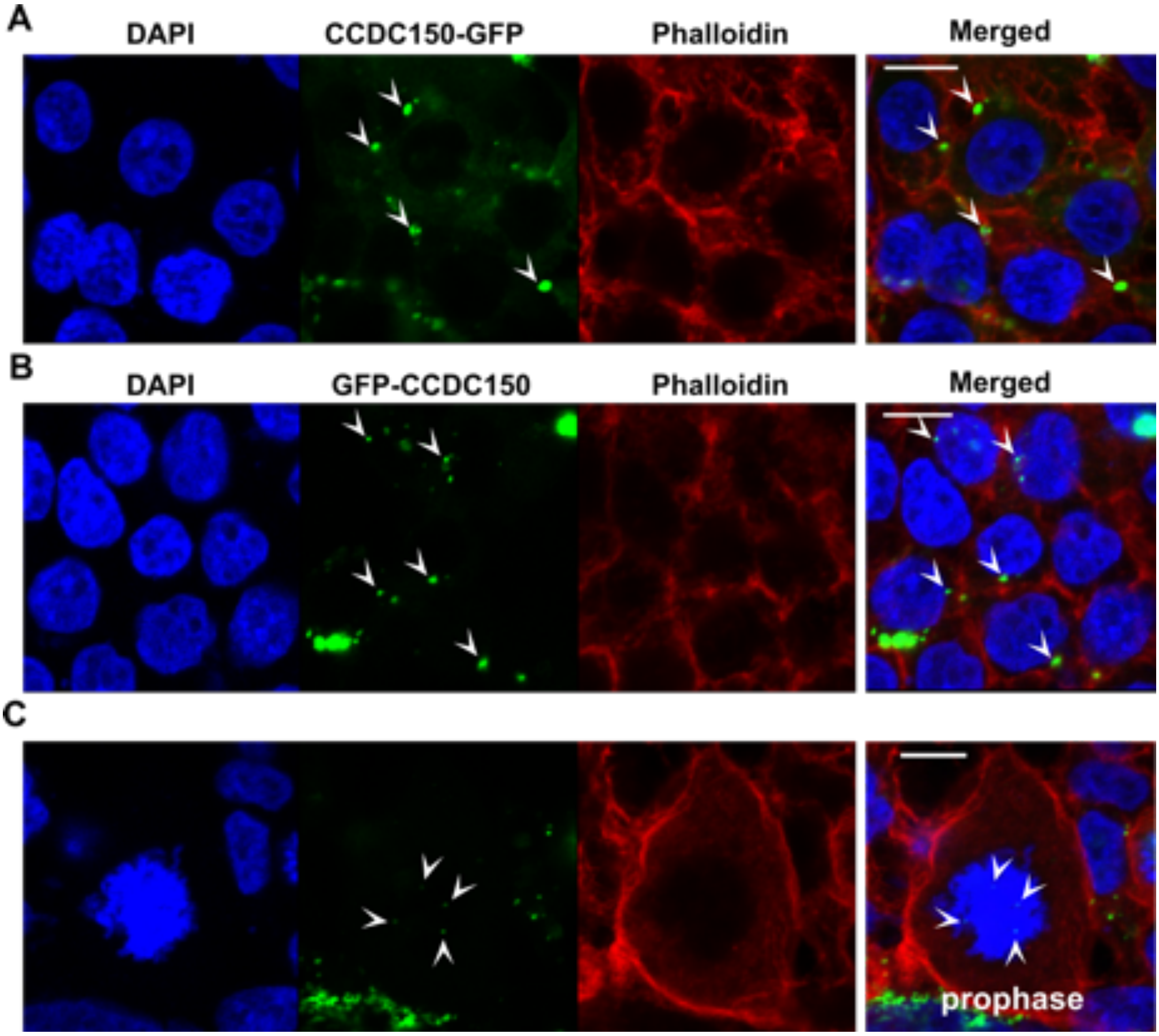

Figure 5. CCDC150-GFP and GFP-CCDC150 expression. (A) Interphase: GFP signal concentrated in perinuclear foci (arrows). (B) Interphase: GFP signal concentrated in perinuclear foci (arrows). (C) Mitotic prophase: GFP signal in punctate foci.

Gene Symbol: *C18orf54*

Gene description: Lung adenoma susceptibility protein 2
C18orf54-GFP: Interphase: GFP signal is concentrated in a single perinuclear focus (Figure 6A).
GFP-C18orf54 Interphase: GFP signal is concentrated in a single perinuclear focus (Figure 6B). Mitotic anaphase: GFP signal localised with the mitotic spindle and cytoplasm (Figure 6C).
-N vs C- GFP expression: Consistent.

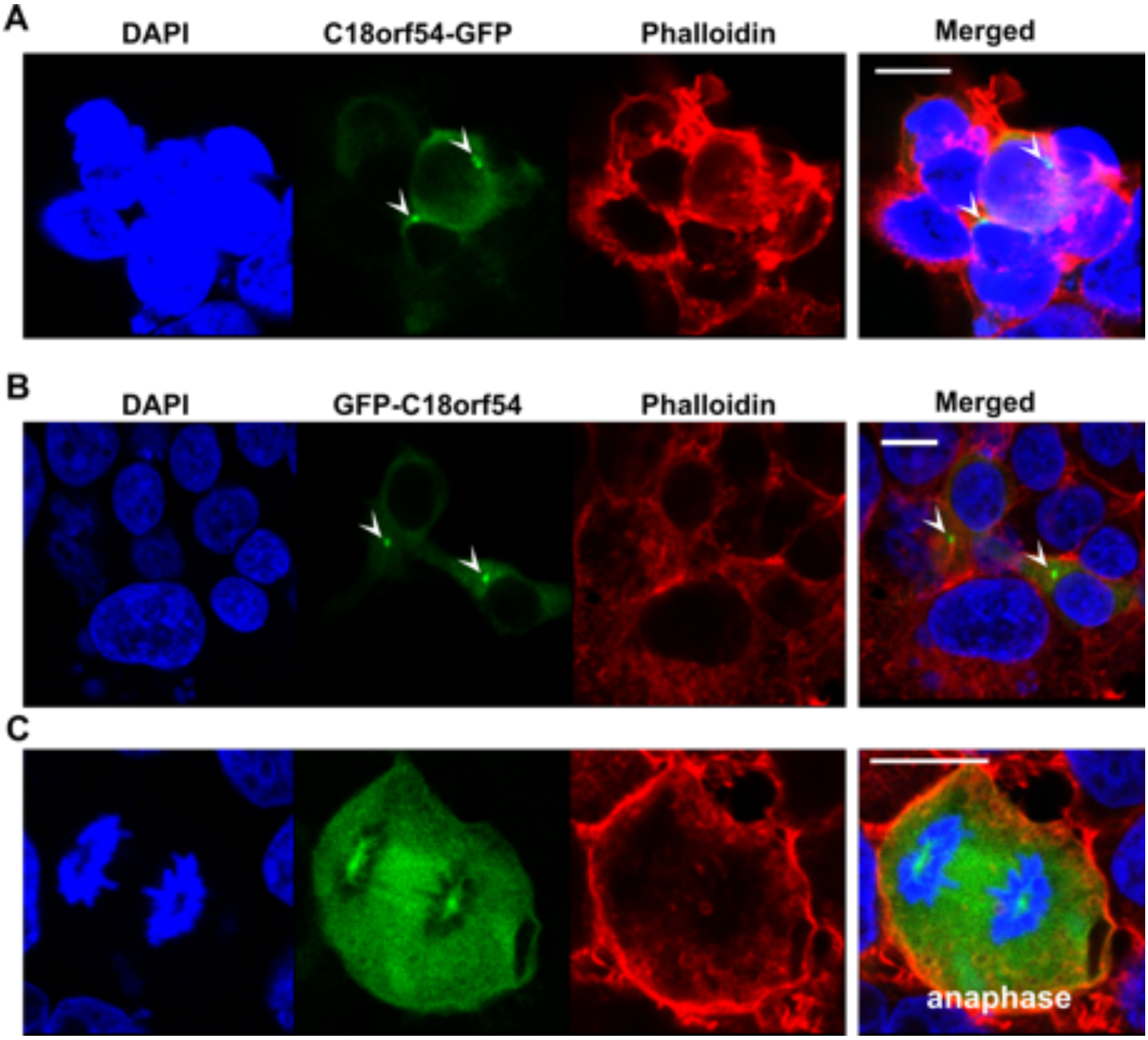

Figure 6 C18orf54-GFP and GFP-C18orf54 expression. (A) GFP signal is concentrated in a single perinuclear focus (arrows). (B) GFP signal is concentrated in a single perinuclear focus (arrows). (C) GFP signal localised with the mitotic spindle and cytoplasm.

Gene Symbol: ***C9orf40***

Gene description: uncharacterised protein C9orf40
C9orf40-GFP: Interphase: GFP signal shows a punctuated pattern in the nucleus (Figure 7A). Mitotic prometaphase: GFP signal concentrated in two loci proximal to DNA (Figure 7B-C).
GFP-C9orf40: Interphase: punctuated and diffuse GFP signal in the nucleus (Figure 8A-B respectively). Mitotic prophase: GFP signal diffuse in cytoplasm (Figure 8C).
-N vs C- GFP expression: Consistent.

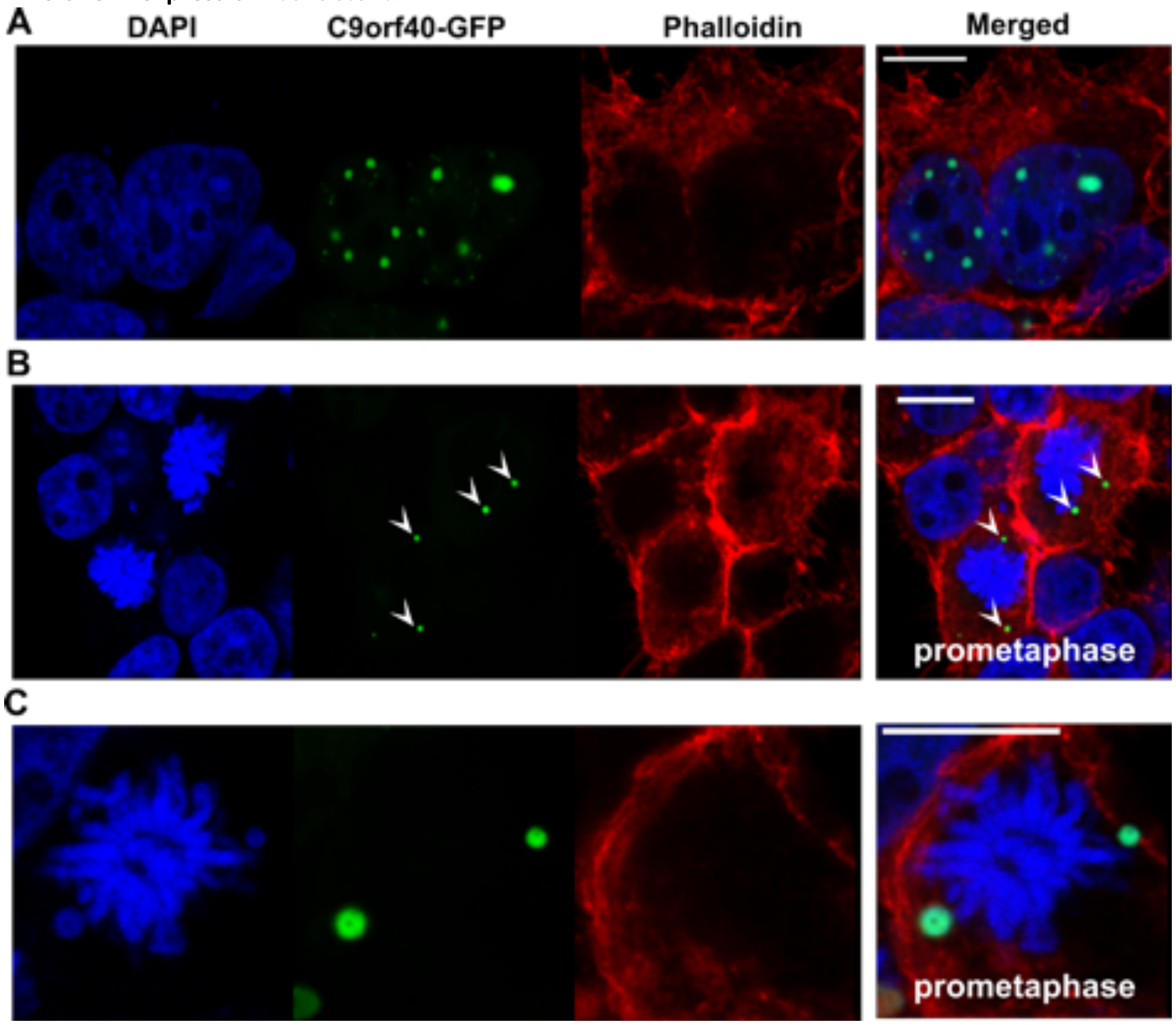

Figure 7. Expression of C9orf40-DEST47 in HEK293T cells. (A) GFP signal shows a punctuated pattern in the nucleus. (B-C) GFP signal concentrated in two loci proximal to DNA. Nuclei were stained with DAPI (blue), uncharacterised proteins were fused with GFP (green) and actin was stained with phalloidin and Texas Red (red). Scale bars = 10 µm

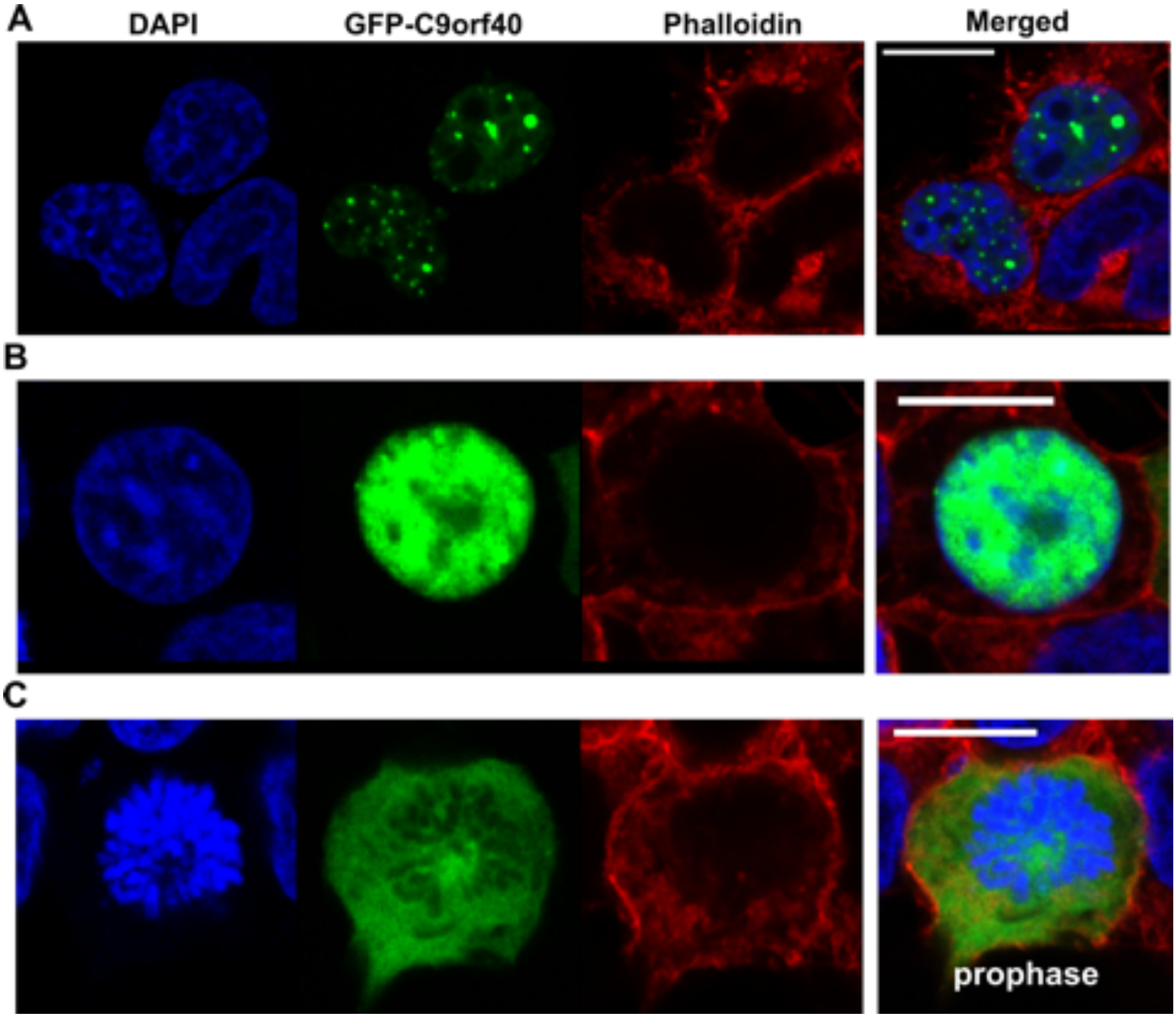

Figure 8. Expression of C9orf40-DEST53 in HEK293T cells. (A) Expression of C9orf40-DEST53 was found with similar pattern of C9orf40-DEST47, although also a diffuse expression in nuclei but not nucleoli was seen (B). (C) Expression of C9orf40-DEST53 showed diffuse expression during prophase/prometaphase. Nuclei were stained with DAPI (blue), uncharacterised proteins were fused with GFP (green) and actin was stained with phalloidin and Texas Red (red). Scale bars= 10 µm

Gene Symbol: ***ERI2***

Gene description: ERI1 exoribonuclease family member 2
ERI2-GFP: No signal.
GFP-ERI2: Interphase: GFP signal is concentrated in a single perinuclear locus (Figure 9A) and diffused in cytoplasm (Figure 9B). Mitotic prophase: GFP signal is diffused in cytoplasm (Figure 9C).
-N vs C- GFP expression: Not confirmed.

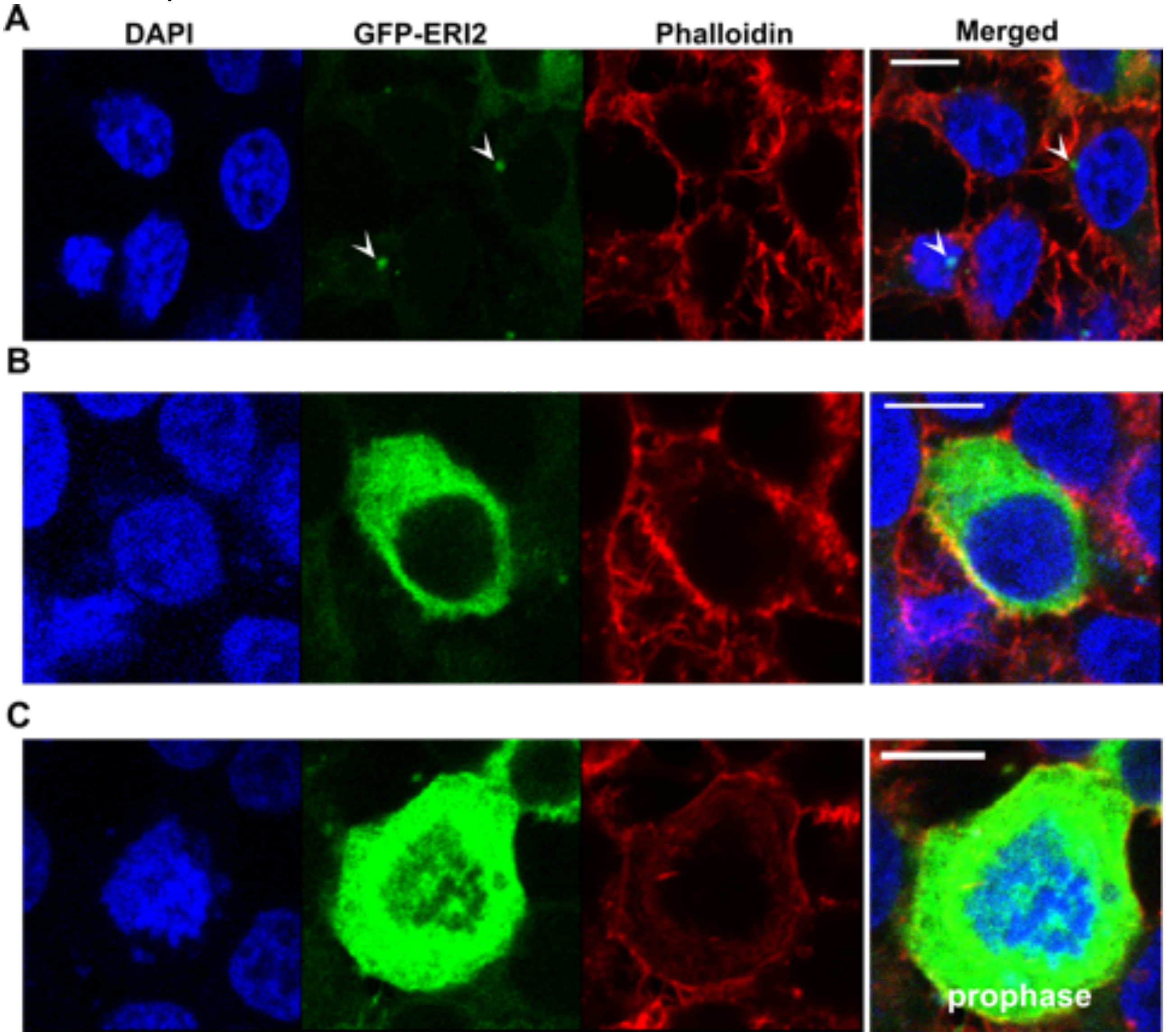

Figure 9. GFP-ERI2 expression. (A) Interphase: GFP signal is concentrated in a single perinuclear locus (arrows). (C) Mitotic prophase: GFP signal is diffused in cytoplasm.

Gene Symbol: ***ZNF695***

Gene description: zinc finger protein 695
ZNF695-GFP: No GFP signal.
GFP-ZNF695: Interphase: GFP signal is concentrated in perinuclear aggregates (Figure 10A). Mitotic prophase: Punctuated GFP signal (Figure 10B).
-N vs C- GFP expression: Not confirmed.

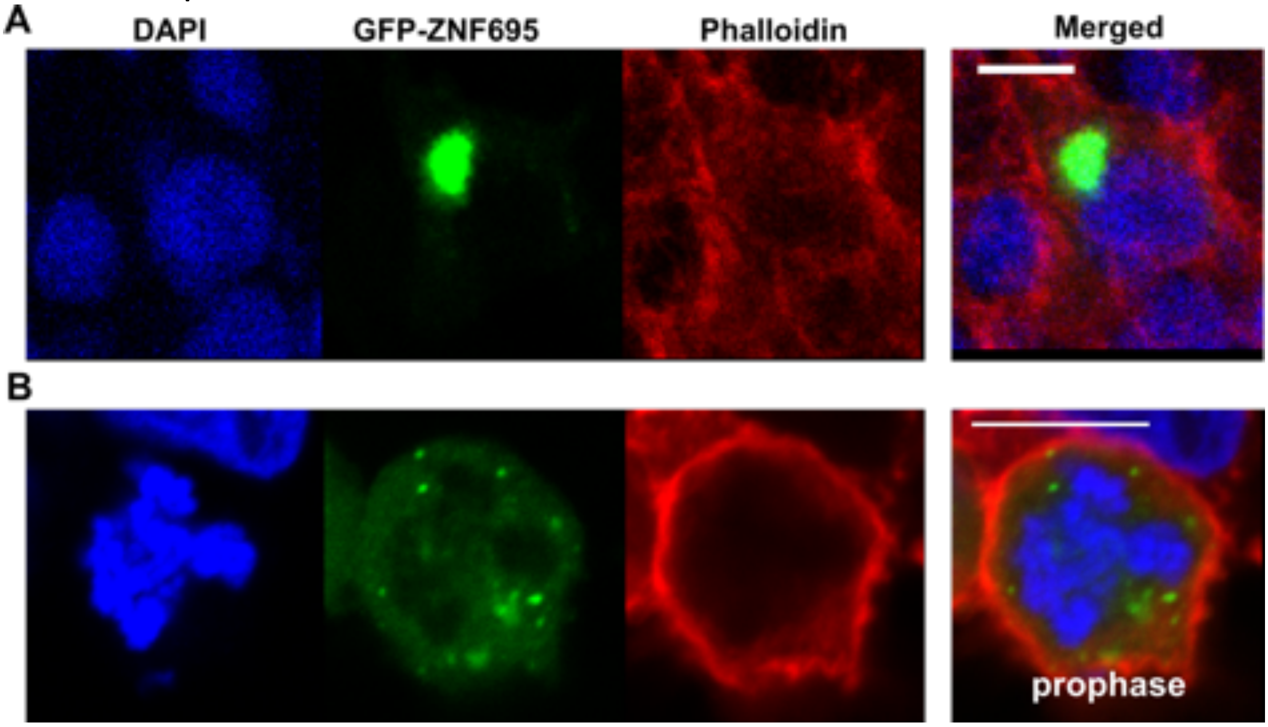

Figure 10. Expression of ZNF695-DEST53 in HEK293T cells. (A) GFP signal is concentrated in perinuclear aggregates. (B) Mitotic prophase: Punctuated GFP signal.

Gene Symbol: ***ZNF100***

Gene description: zinc finger protein 100
ZNF100-GFP: GFP signal is diffused in cytoplasm and nucleus but no nucleoli (Figure 11A).
GFP-ZNF100: GFP signal is diffused and focused in aggregates in cytoplasm (Figure 11B). Mitotic prophase: GFP signal is diffused and focused in aggregates in cytoplasm (Figure 11C).
-N vs C- GFP expression: Partial agreement.

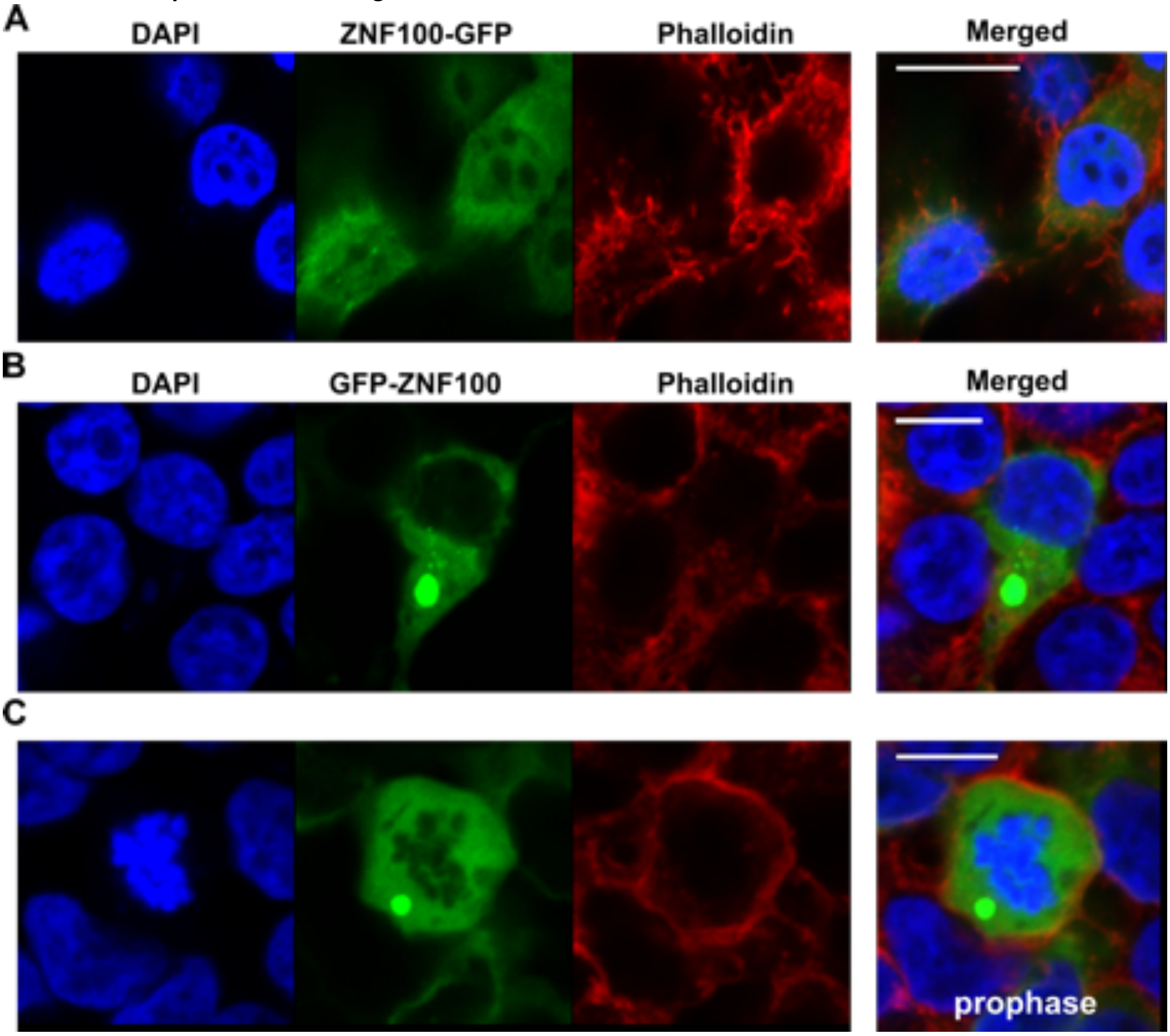

Figure 11. Expression of ZNF100-DEST47/53 in HEK293T cells. (A) GFP signal is diffused in cytoplasm and nucleus but no nucleoli. (B) GFP signal is diffused and focused in aggregates in cytoplasm. (C). Mitotic prophase: GFP signal is diffused and focused in aggregates in cytoplasm

Gene Symbol: ***C19orf48***

Gene description: chromosome 19 open reading frame 48
C19orf48-GFP: No GFP signal.
GFP-C19orf48: Mitotic prometaphase: GFP signal focused in two loci proximal to condensed chromatin (Figure 12).
-N vs C- GFP expression: Not confirmed.

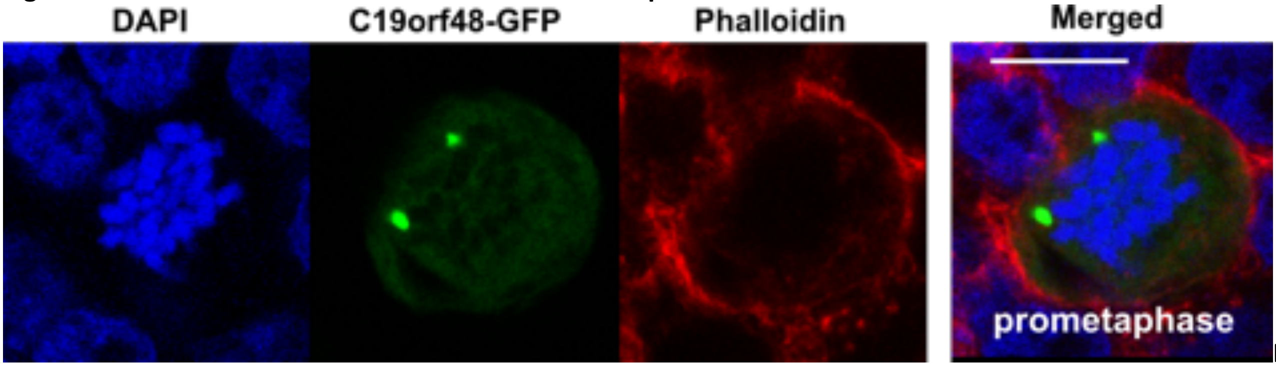

Figure 12. Expression of C19orf48-DEST47 in HEK293T cells. Mitotic prometaphase: GFP signal focused in two loci proximal to condensed DNA.

Gene Symbol: ***C17orf53***

Gene description: uncharacterised protein C17orf53

C17orf53-GFP: Interphase: Punctuate GFP signal concentrated in nucleus but not nucleoli (Figure 13A). Mitotic prophase: GFP signal diffuse in the cytoplasm (Figure 13B).

GFP-C17orf53: Interphase GFP signal is concentrated in nucleus and in large aggregates in perinuclear region (Figure 13C).

-N vs C- GFP expression: Partial agreement.

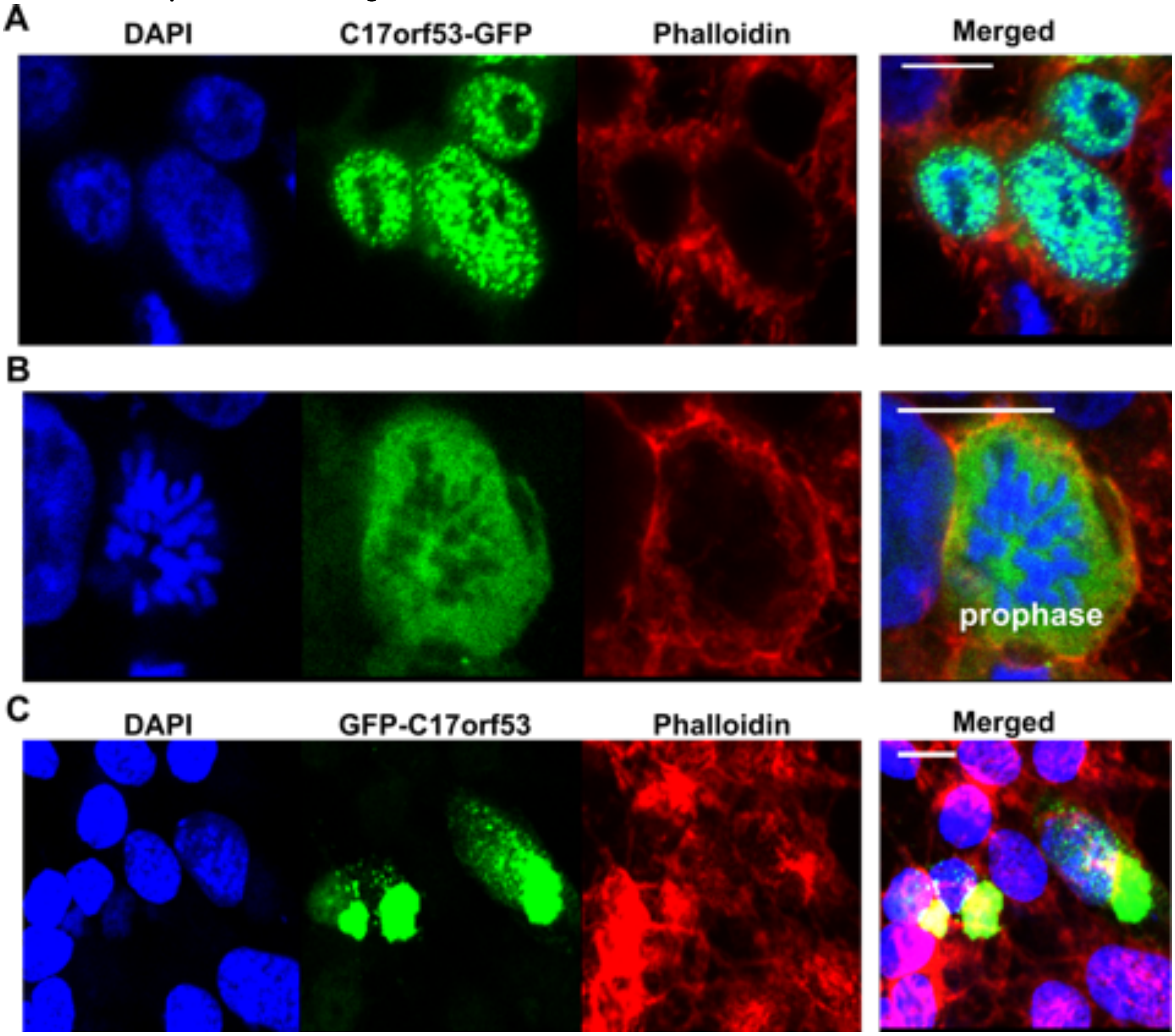

Figure 13. C17orf53-GFP and GFP-C17orf53 expression. (A): Interphase: Punctuate GFP signal in nucleus but not nucleoli. (B) Mitotic prophase: GFP signal diffuse in the cytoplasm. (C) Interphase GFP signal is concentrated in nucleus and in large aggregates in perinuclear region.

Gene Symbol: *DONSON*

Gene description: downstream neighbour of Son
DONSON-GFP: Interphase: Punctuate GFP signal is concentrated in nucleus (Figure 14).
GFP-DONSON: Interphase: Punctuate GFP signal is concentrated in nucleus (Figure 15A). Mitotic prometaphase: GFP signal focused in one focus (Figure 15B). Mitotic prometaphase: GFP signal focused in two foci proximal to condensed DNA. (Figure 15C).
-N vs C- GFP expression: Consistent.

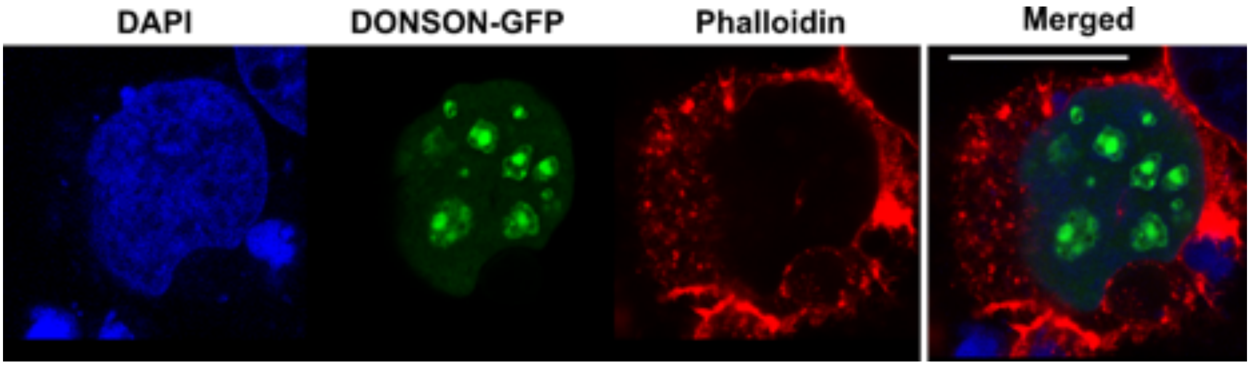

Figure 14. DONSON-GFP expression. (A) Expression of DONSON-DEST47 stained the nuclei with the inclusion of nucleoli.

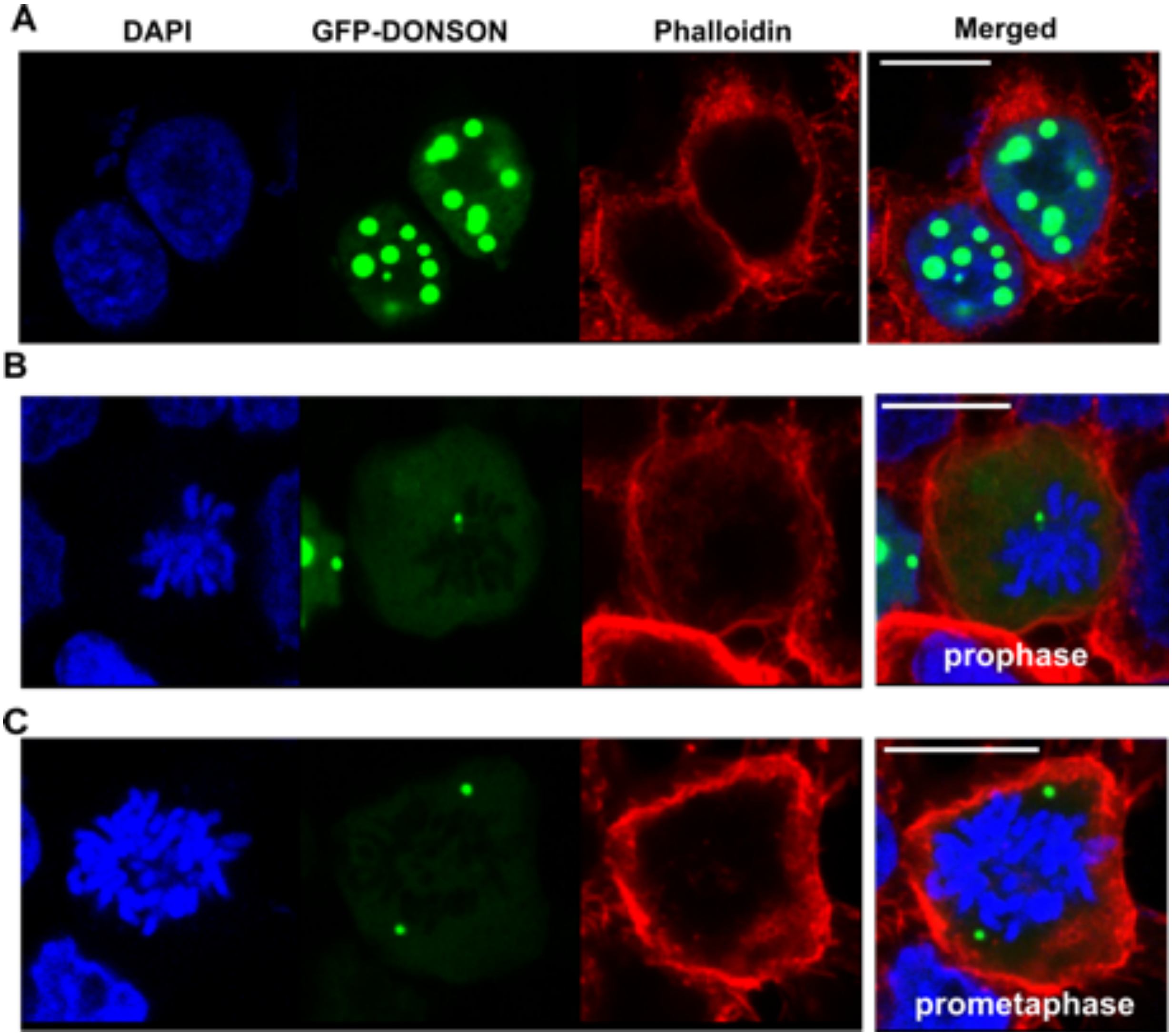

Figure 15. GFP-DONSON expression. (A) Interphase: Punctuate GFP signal is concentrated in nucleus. (B) Mitotic prometaphase: GFP signal focused in one focus. (C) Mitotic prometaphase: GFP signal focused in two foci proximal to condensed.

Gene Symbol*: WDR76*

Gene description*:* WD repeat-containing protein 76
WDR76-GFP: Interphase: GFP signal is diffuse in nuclei but not nucleoli (Figure 16).
GFP-WDR76: No GFP signal.
-N vs C- GFP expression: Not confirmed.

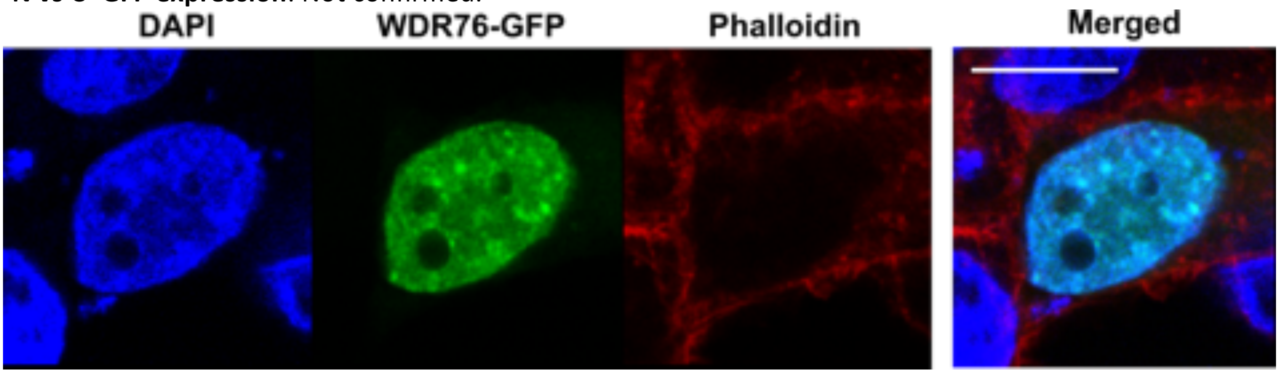

Figure 16. WDR76-GFP expression. : Interphase: GFP signal is diffuse in nuclei but not nucleoli.

Gene Symbol: ***MTFR2***

Gene description: mitochondrial fission regulator 2
MTFR2-GFP: No GFP signal.
GFP-MTFR2: Interphase: Punctuate GFP signal in nucleus and nucleoli (Figure 17A). Mitotic prophase: Punctuate GFP signal (Figure 17B).
-N vs C- GFP expression: Not confirmed.

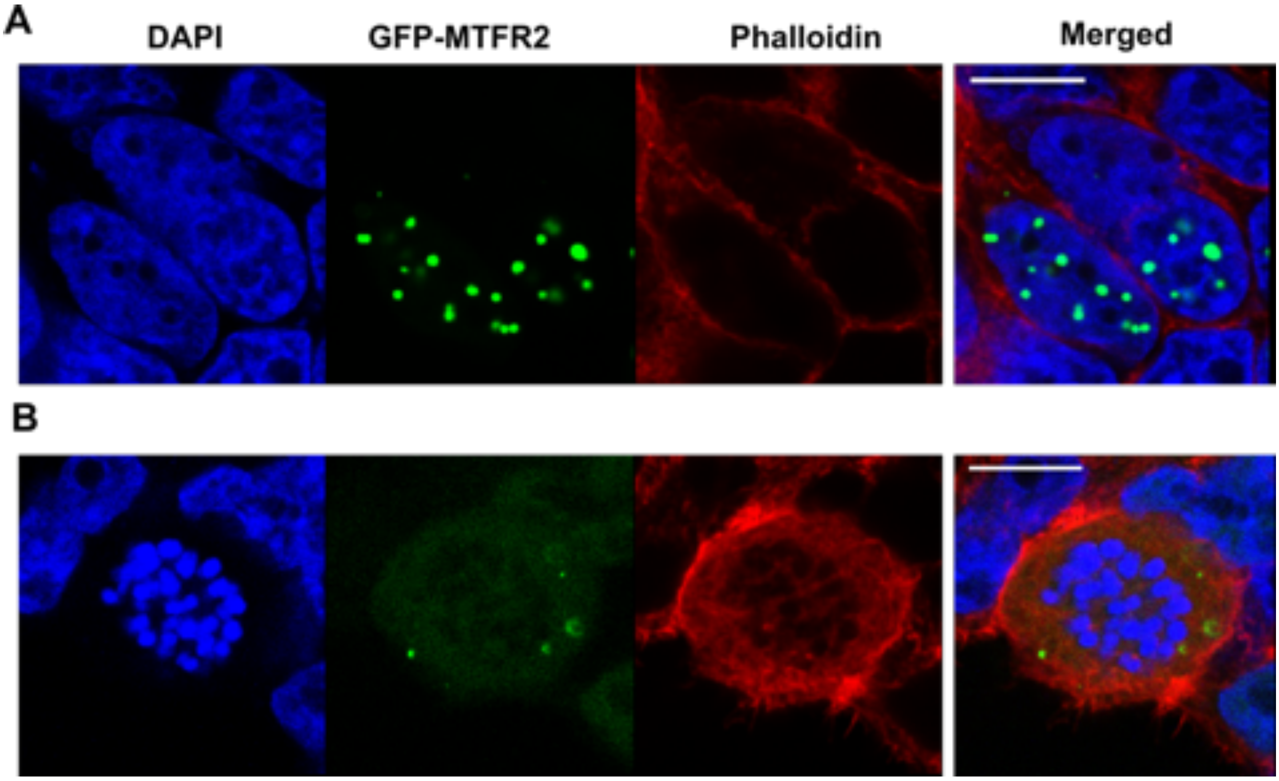

Figure 17. GFP-MTFR2 expression. (A) Interphase: Punctuate GFP signal in nucleus and nucleoli. (B Mitotic prophase: Punctuate GFP signal.

Gene Symbol: ***CCDC34***

Gene description: *Coiled-coil domain-containing protein 34*

CCDC34-GFP: Interphase: GFP signal diffuse in cytoplasm and concentrated in perinuclear region (Figure 18A).

GFP-CCDC34: Interphase: GFP signal diffuse in cytoplasm (Figure 18B). Mitotic metaphase: GFP signal diffuse in cytoplasm (Figure 18C).

-N vs C- GFP expression: Partial agreement.

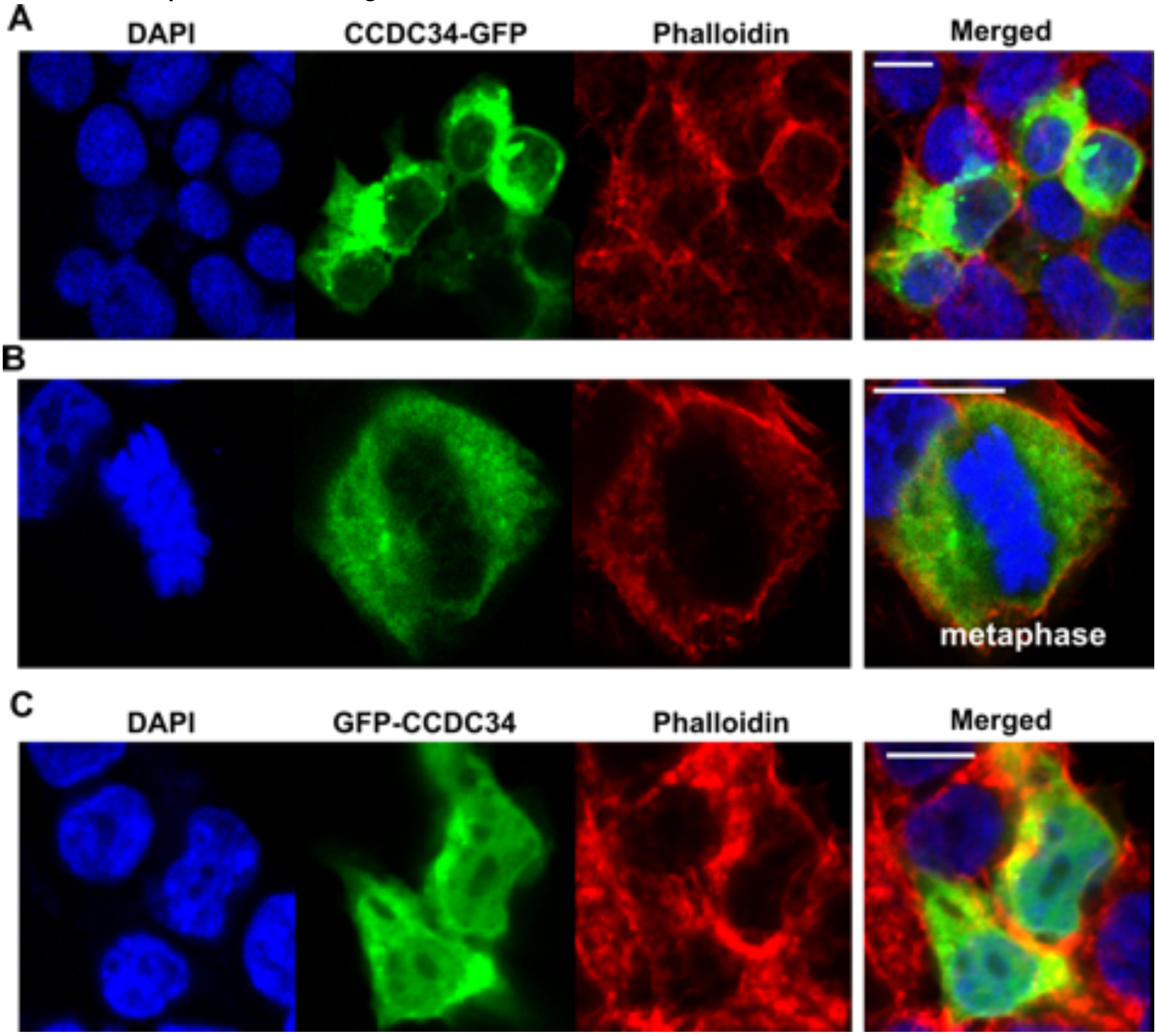

Figure 18. CCDC34-GFP and GFP-CCDC34 expression. (A) Interphase: GFP signal diffuse in cytoplasm and concentrated in perinuclear region. (B) Interphase: GFP signal diffuse in cytoplasm. (C) Mitotic metaphase: GFP signal diffuse in cytoplasm.

Gene Symbol: ***FAM122B***

Gene description: family with sequence similarity 122B

FAM122B-GFP: Interphase: GFP signal is diffuse in nuclei but not nucleoli (Figure 19A). Mitotic prophase: GFP signal is diffuse in cytoplasm (Figure 19B)

GFP-FAM122B*:* Interphase: GFP signal is diffuse in nuclei but not nucleoli (Figure 19C). Mitotic prophase: GFP signal is diffuse in cytoplasm (Figure 19D)

-N vs C- GFP expression*:* Consistent.

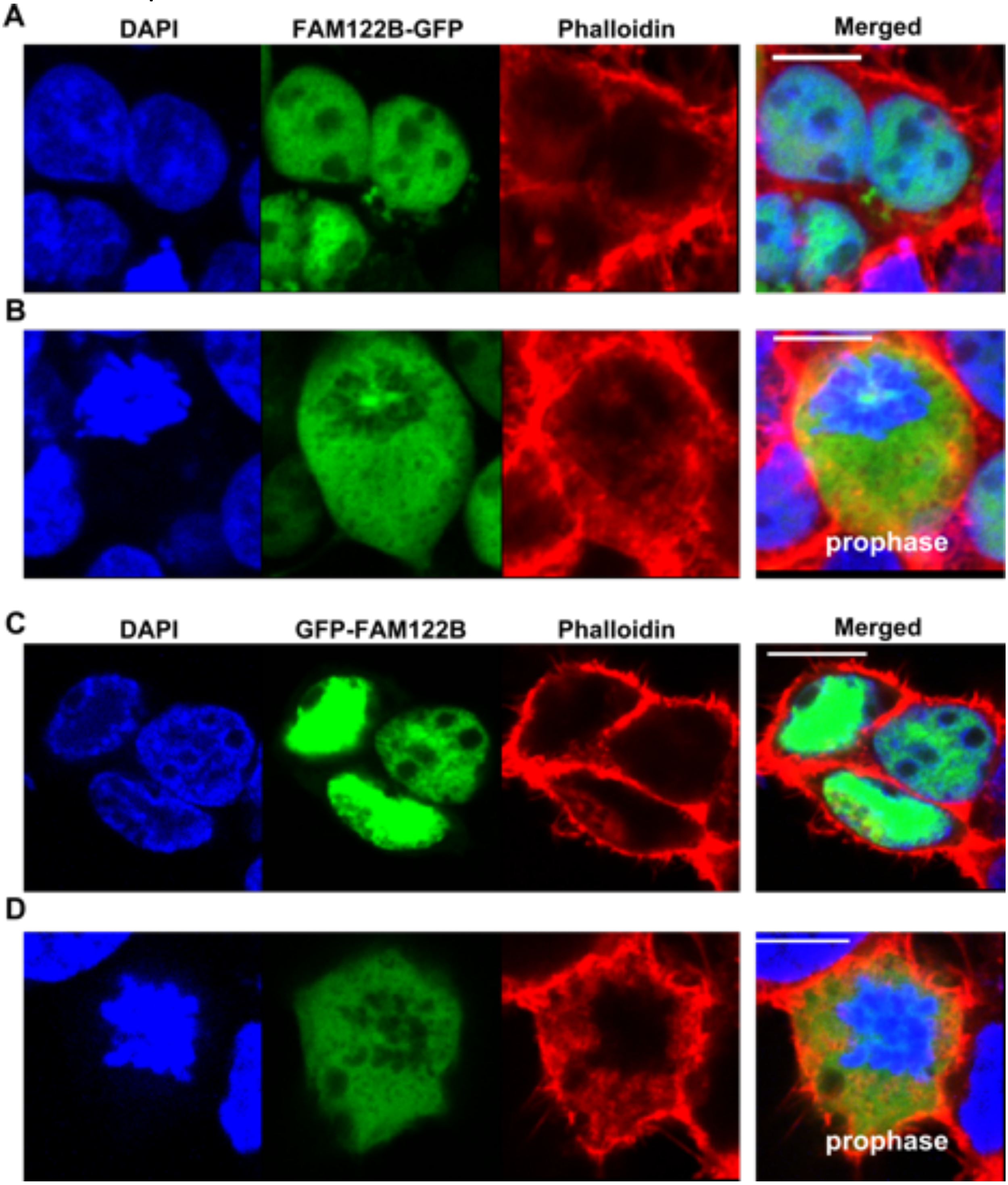

Figure 19. FAM122B-GFP and GFP-FAM122B expression. (A) Interphase: GFP signal is diffuse in nuclei but not nucleoli. (B) Mitotic prophase: GFP signal is diffuse in cytoplasm. (C) Interphase: GFP signal is diffuse in nuclei but not nucleoli. (D) Mitotic prophase: GFP signal is diffuse in cytoplasm.

Gene Symbol*: SPIN4*

Gene description*:* spindlin family member 4
SPIN4-GFP: Interphase: GFP signal concentrated in nuclear bodies and nucleoli (Figure 20A).
GFP-SPIN4: Interphase: GFP signal concentrated in nuclear bodies and nucleoli (Figure 20B).
-N vs C- GFP expression*:* Consistent.

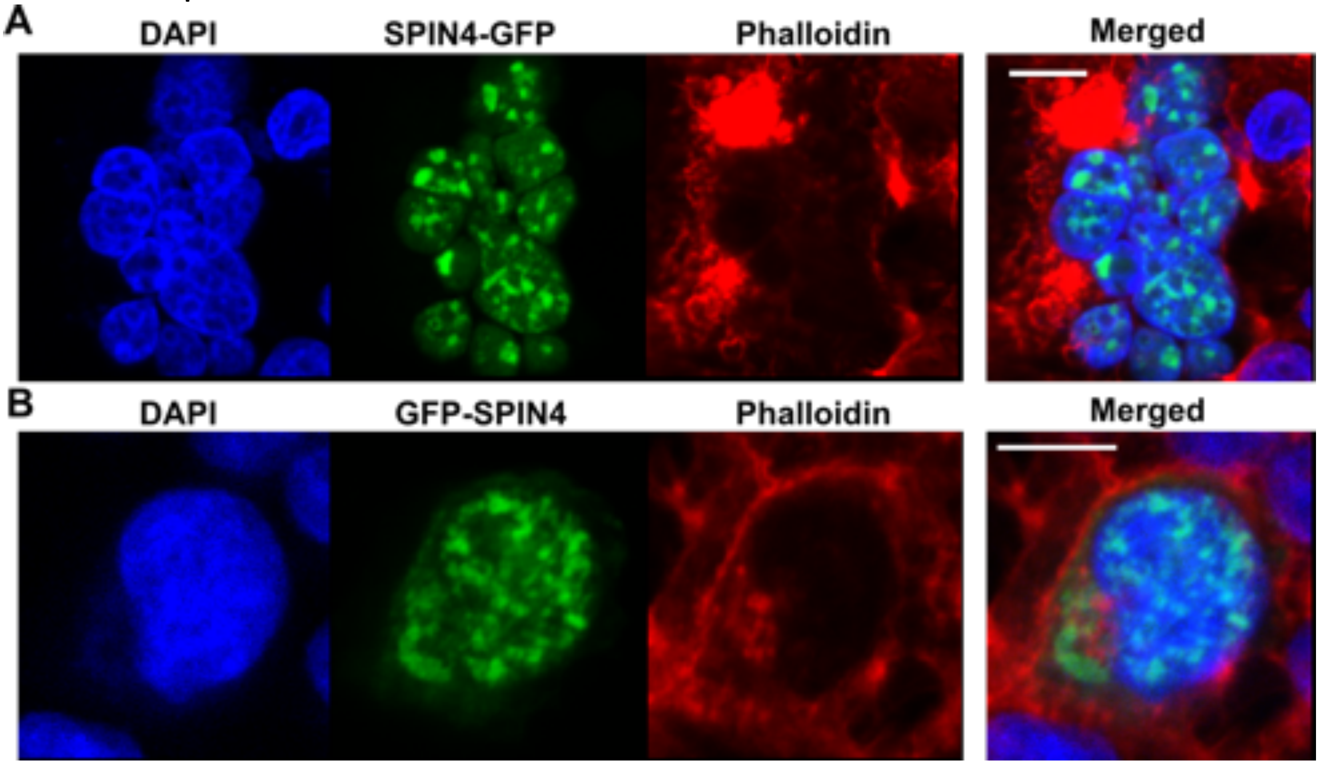

Figure 20. SPIN4-GFP and GFP-SPIN4 expression. (A) Interphase: GFP signal concentrated in nuclear bodies and nucleoli. (B) Interphase: GFP signal concentrated in nuclear bodies and nucleoli.

Gene Symbol: *CHAC2*

Gene description: ChaC cation transport regulator homolog 2
CHAC2-GFP: Interphase: GFP signal diffuse in cytoplasm and nuclei (Figure 21A). Mitotic anaphase: GFP signal diffuse in cytoplasm (Figure 21B).
GFP-CHAC2: Interphase: GFP signal diffuse in cytoplasm and nuclei (Figure 21C). Mitotic metaphase: GFP signal diffuse in cytoplasm (Figure 21D).
-N vs C- GFP expression: Consistent.

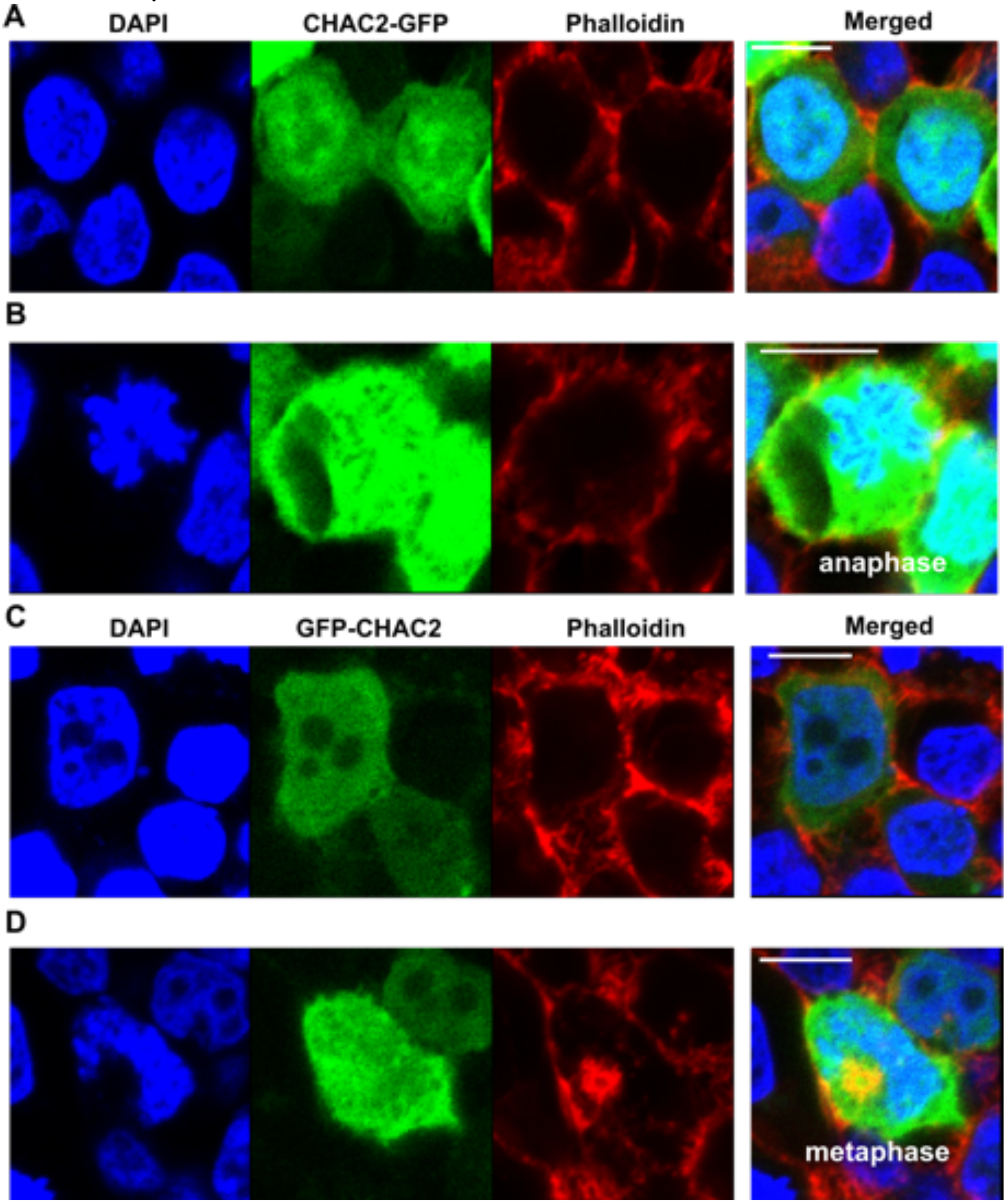

Figure 21. CHAC2-GFP and GFP-CHAC2 expression. (A) Interphase: GFP signal diffuse in cytoplasm and nuclei. (B) Mitotic anaphase: GFP signal diffuse in cytoplasm. (C) Interphase: GFP signal diffuse in cytoplasm and nuclei (D). Mitotic metaphase: GFP signal diffuse in cytoplasm.

Gene Symbol: ***EZH2***

Gene description: enhancer of zeste 2 polycomb repressive complex 2 subunit
EZH2-GFP: Interphase: GFP signal diffuse in nuclei and nucleoli (Figure 22A).
GFP-EZH2: Interphase: GFP signal diffuse in nuclei and nucleoli (Figure 22B). Mitotic prophase: GFP signal diffuse in cytoplasm (Figure 22C).
-N vs C- GFP expression: Consistent.

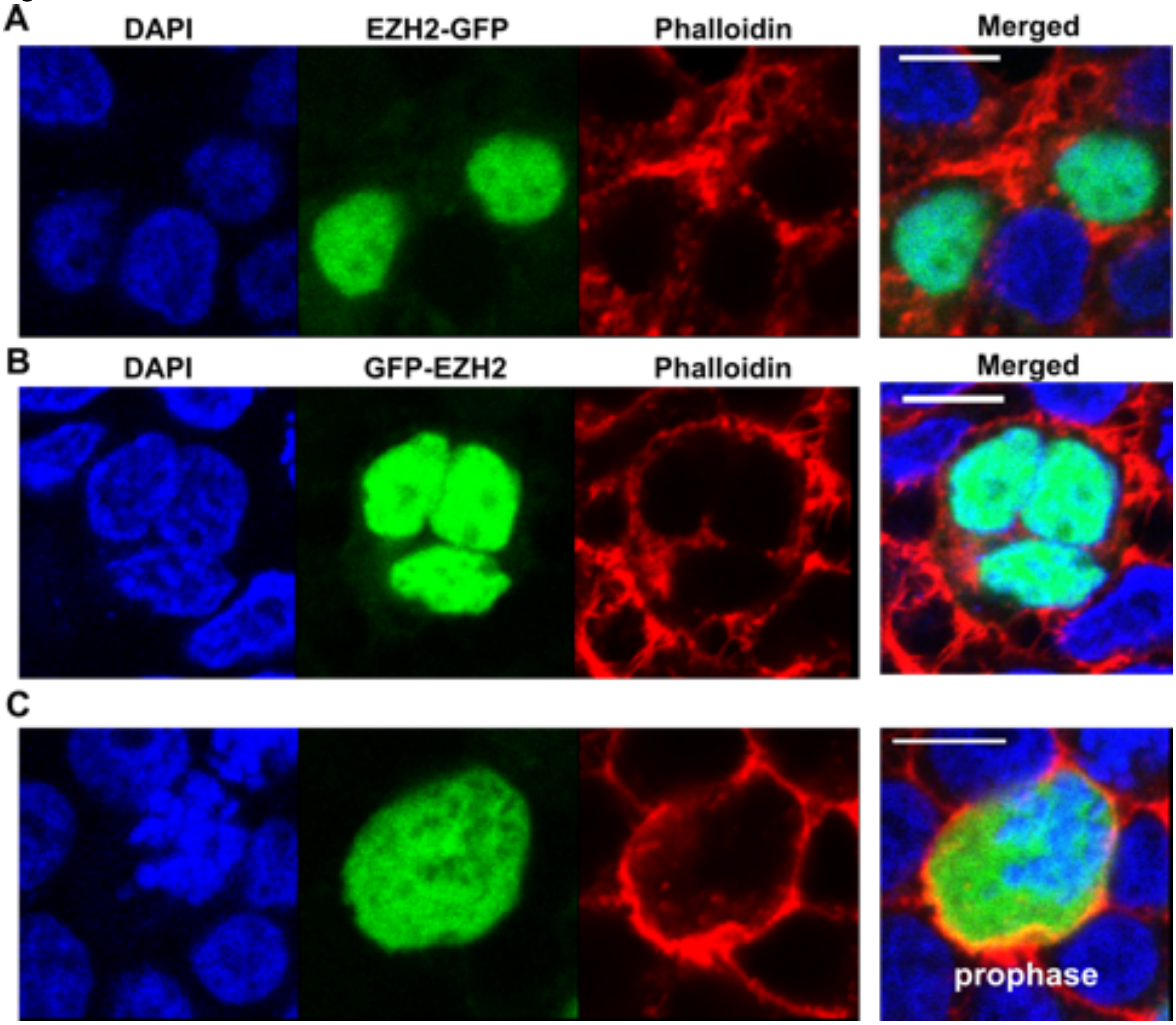

Figure 22. EZH2-GFP and GFP-EZH2 expression. (A) Interphase: GFP signal diffuse in nuclei and nucleoli. (B) Interphase: GFP signal diffuse in nuclei and nucleoli. (C) Mitotic prophase: GFP signal diffuse in cytoplasm.

Gene Symbol: ***UBR7***

Gene description: ubiquitin protein ligase E3 component n-recognin 7 (putative)
UBR7-GFP: Interphase: GFP signal diffuse in nuclei but not nucleoli (Figure 23A). Mitotic metaphase: GFP signal diffuse in cytoplasm and concentrated on mitotic spindle (Figure 23B).
GFP-UBR7: Interphase: GFP signal diffuse in nuclei but not nucleoli (Figure 23C). Mitotic metaphase: GFP signal diffuse in cytoplasm (Figure 23D).
-N vs C- GFP expression: Consistent.

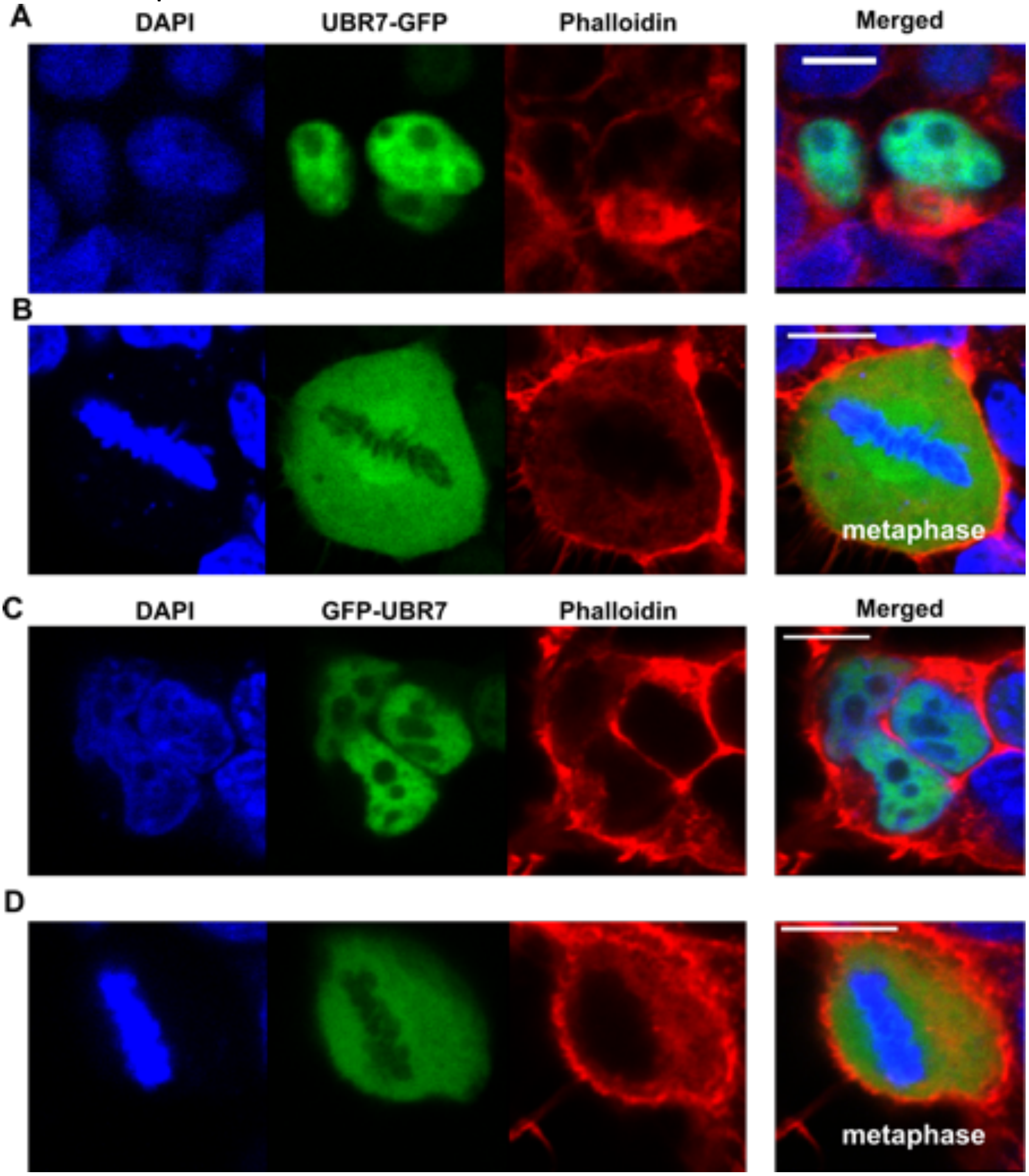

Figure 23. UBR7-GFP and GFP-UBR7 expression. (A) Interphase: GFP signal diffuse in nuclei but not nucleoli. (B) Mitotic metaphase: GFP signal diffuse in cytoplasm and concentrated on mitotic spindle. (C) Interphase: GFP signal diffuse in nuclei but not nucleoli. (D) Mitotic metaphase: GFP signal diffuse in cytoplasm.

Gene symbol: ***KIAA1524***

Gene description: cancerous inhibitor of protein phosphatase 2A, CIP2A
KIAA1524-GFP: Interphase: GFP signal is concentrated in aggregates (Figure 24A).
GFP-KIAA1524*:* Interphase: GFP signal is concentrated in aggregates (Figure 24B).
-N vs C- GFP expression: Consistent.

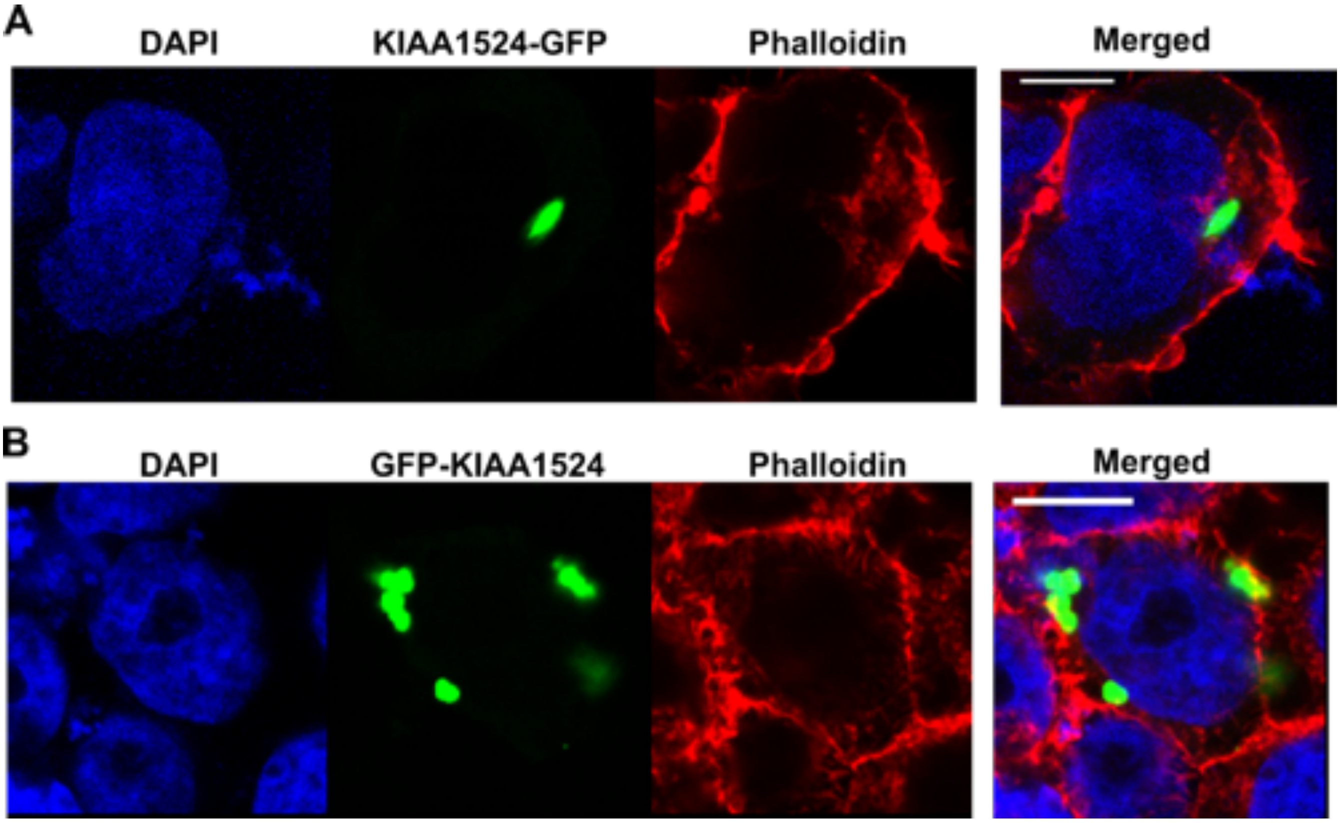

Figure 24. KIAA1524-GFP and GFP-KIAA1524 expression. (A) Interphase: GFP signal is concentrated in aggregates. (B) Interphase: GFP signal is concentrated in aggregates.

Gene Symbol*: RIBC2*

Gene description*:* RIB43A-like with coiled-coils protein 2
RIBC2-GFP*:* Interphase: GFP signal diffuse in cytoplasm and concentrated in perinuclear region (Figure 25A).
GFP-RIBC2*:* Interphase: Punctuated GFP signal and aggregates in cytoplasm (Figure 25B)
-N vs C- GFP expression: Partial.

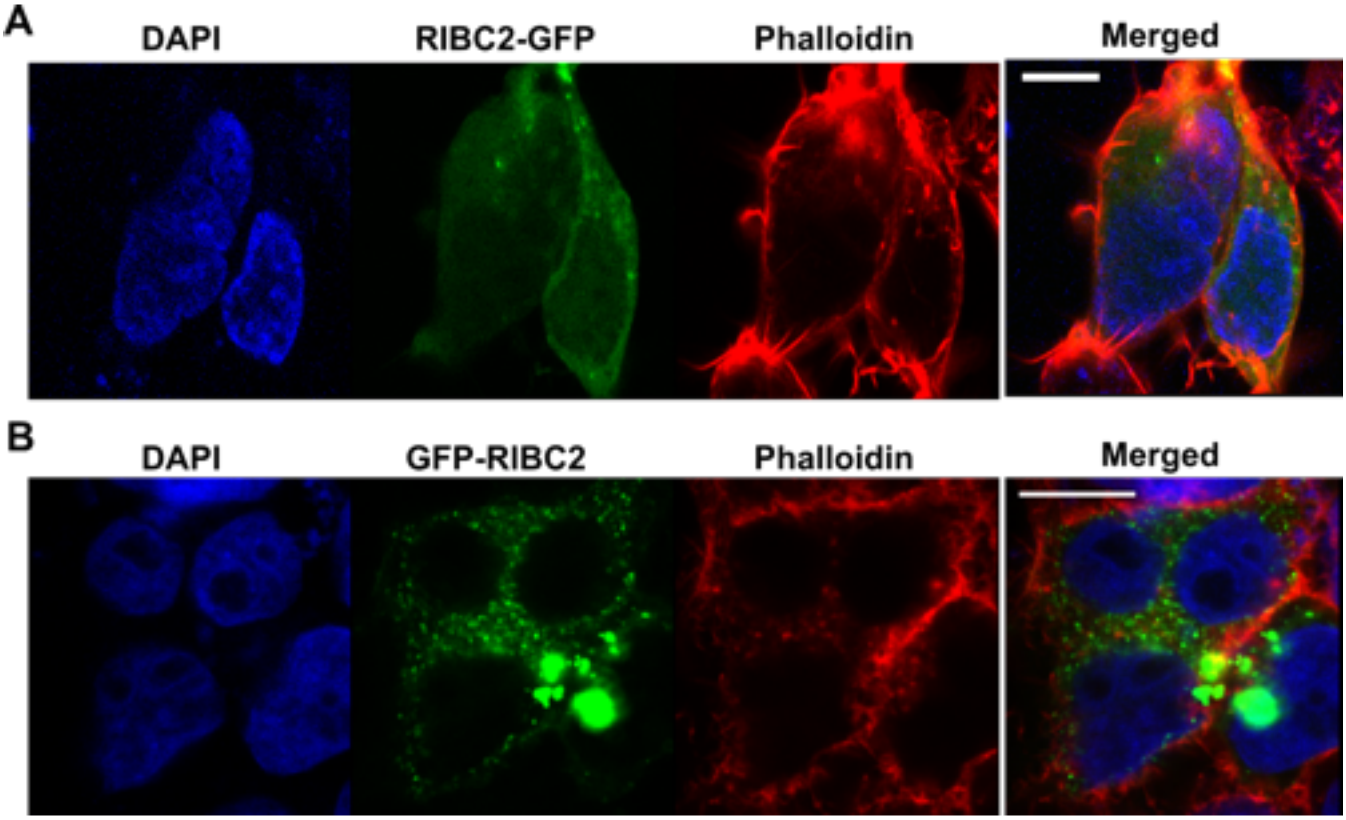

Figure 25.RIBC2-GFP and GFP-RIBC2 expression. (A) Interphase: GFP signal diffuse in cytoplasm and concentrated in perinuclear region. (B) Interphase: Punctuated GFP signal and aggregates in cytoplasm.

Gene Symbol: ***MGME1***

Gene description: Mitochondrial genome maintenance exonuclease 1
MGME1-GFP: Interphase: GFP signal in cytoplasmic aggregates (Figure 26).
GFP-MGME1: No GFP signal.
-N vs C- GFP expression: Not confirmed.

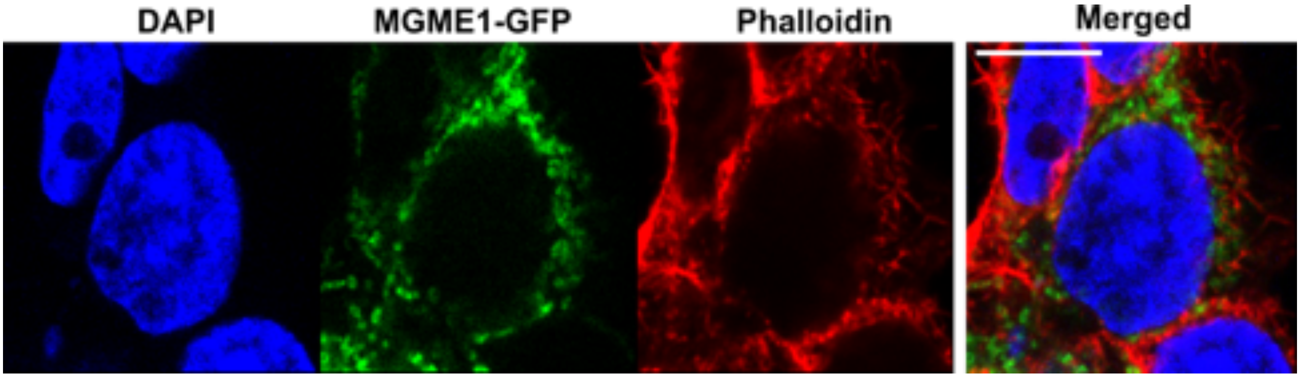

Figure 26. MGME1-GFP expression. Interphase: GFP signal in cytoplas mic aggregates.

Gene Symbol: ***ARHGAP11B***

Gene description: Rho GTPase activating protein 11B
ARHGAP11B-GFP: Interphase: GFP signal in perinuclear aggregates (Figure 27A).
GFP-ARHGAP11B: Interphase: GFP signal in perinuclear aggregates (Figure 27B). Mitotic metaphase: GFP signal diffuse in cytoplasm (Figure 27C).
-N vs C- GFP expression*:* Consistent.

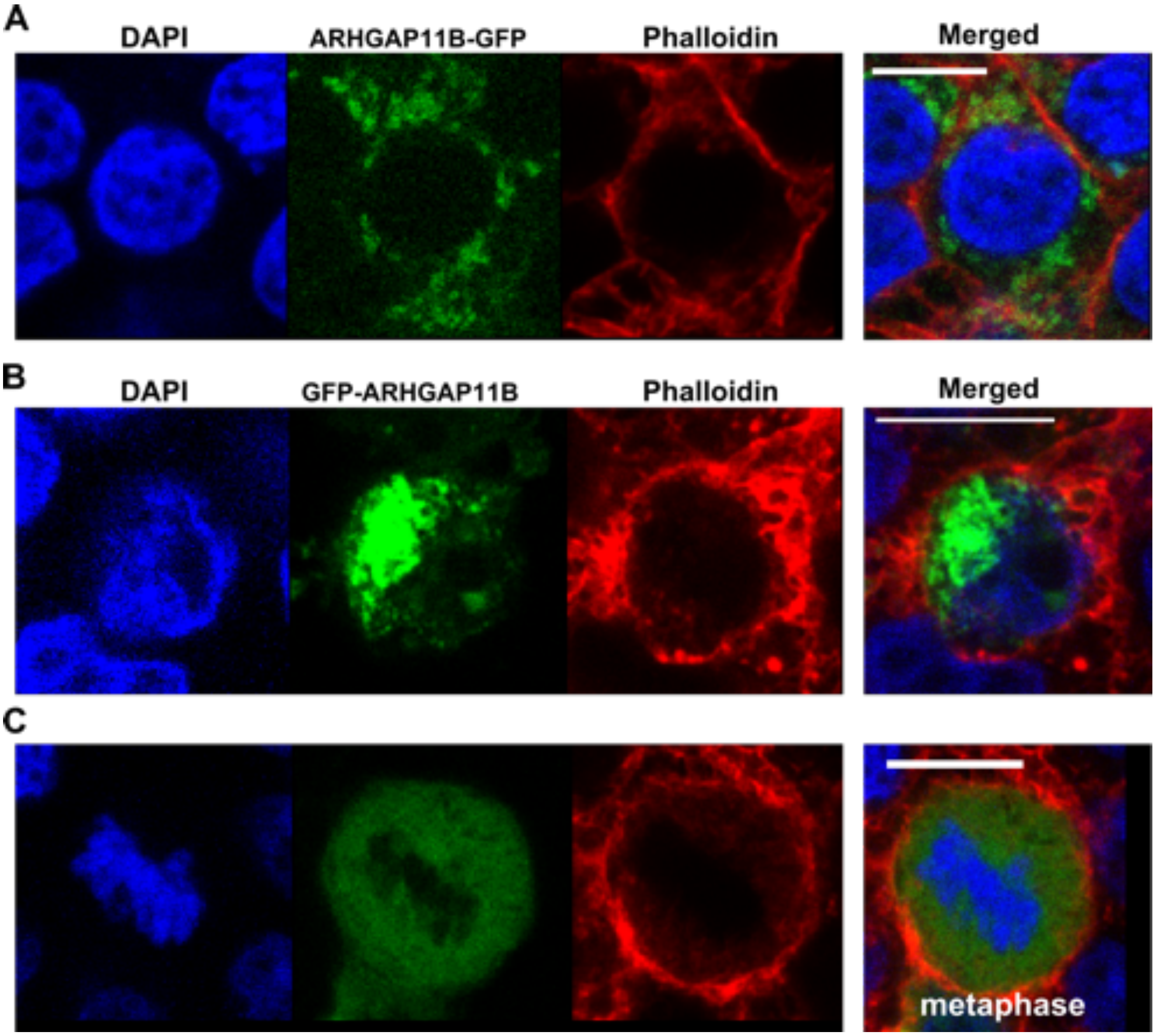

Figure 27 ARHGAP11B-GFP and GFP-ARHGAP11B expression. (A) Interphase: GFP signal in perinuclear aggregates. (B) Interphase: GFP signal in perinuclear aggregates. (C). Mitotic metaphase: GFP signal diffuse in cytoplasm.

Gene Symbol*: ARL6IP6*

Gene description: ADP ribosylation factor like GTPase 6 interacting protein 6
ARL6IP6-GFP: No GFP signal.
GFP-ARL6IP6: Interphase: GFP signal localised on nuclear membrane and cytoplasm (Figure 28A). Mitotic telophase: GFP signal localised on nuclear membrane (Figure 28B).
-N vs C- GFP expression: Not confirmed.

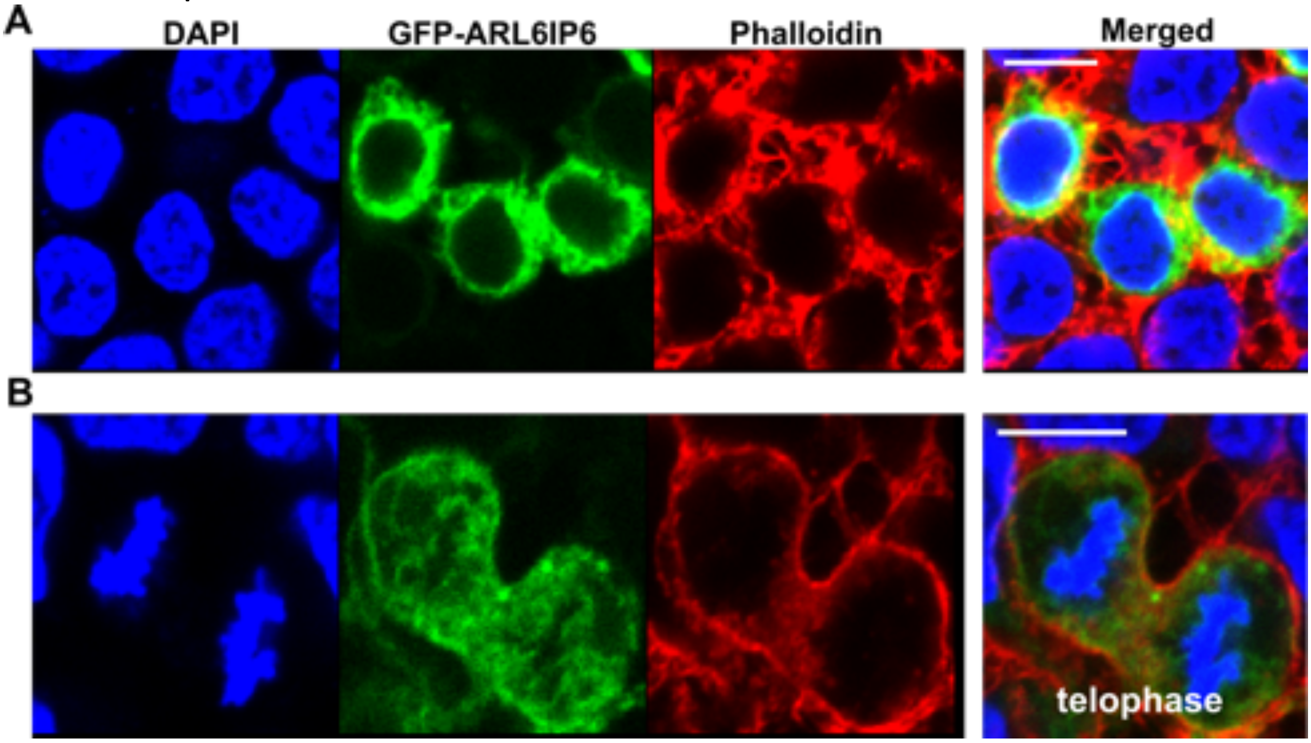

Figure 28. GFP-ARL6IP6 expression. (A) Interphase: GFP signal localised on nuclear membrane and cytoplasm. (B) Mitotic telophase: GFP signal localise on nuclear membrane

Gene Symbol: *TCEANC2*

Gene description: transcription elongation factor A N-terminal and central domain containing 2
TCEANC2-GFP: No GFP signal.
GFP-TCEANC2: Interphase: GFP signal is diffuse in nuclei and focused in nuclear bodies (Figure 29A/B). Mitotic prophase: Punctuated GFP signal proximal to condensed DNA (Figure 29C).
-N vs C- GFP expression: Not confirmed.

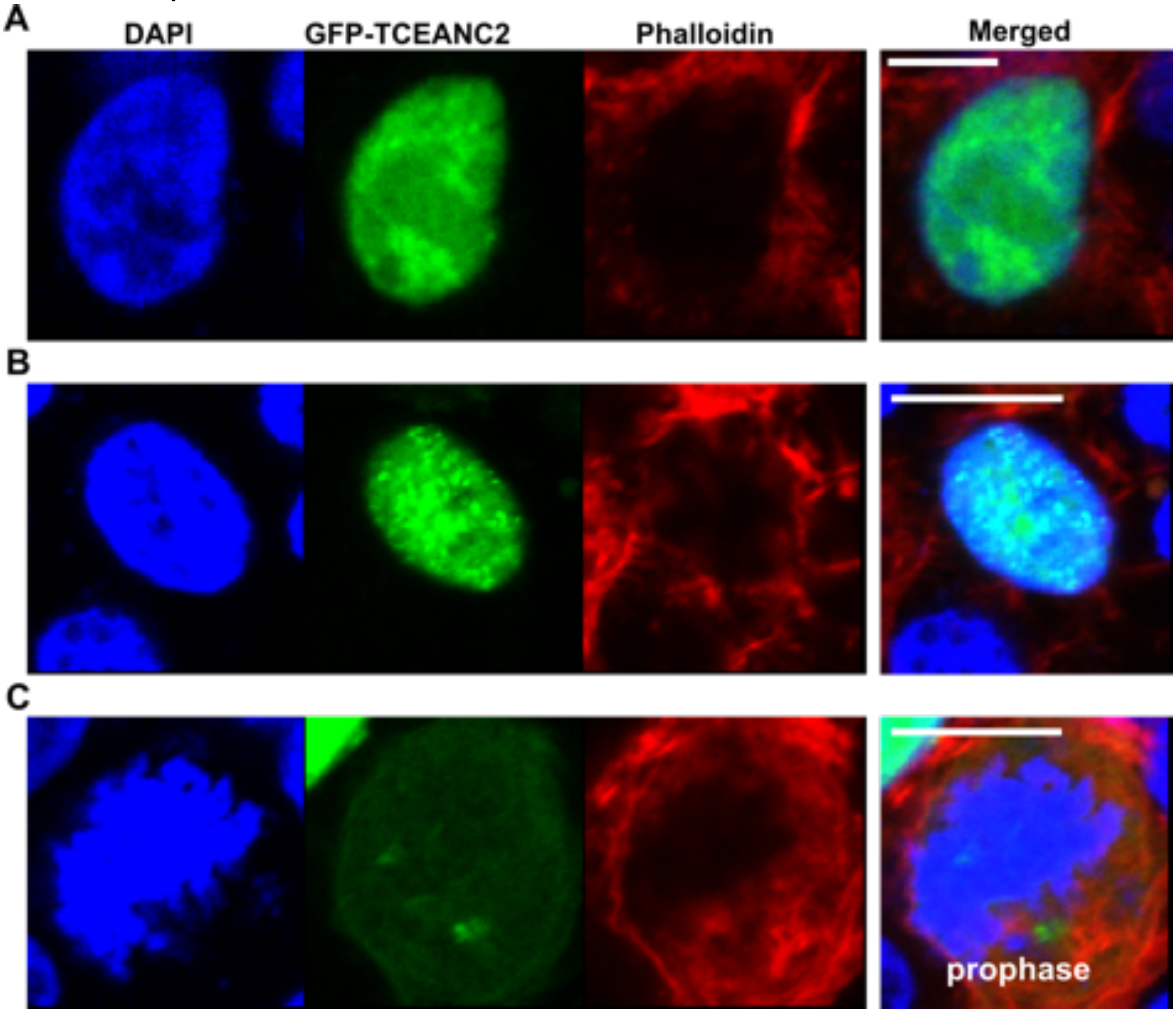

Figure 29. GFP-TCEANC2 expression. (A) Interphase: GFP signal is diffuse in nuclei (A) and focused in nuclear bodies (B). (C) Mitotic prophase: Punctuated GFP signal proximal to condensed DNA.

Gene Symbol: ***TMEM106C***

Gene description: transmembrane protein 106C
TMEM106C-GFP: No GFP signal.
GFP-TMEM106C: Interphase: GFP signal localised on nuclear membrane and cytoplasmic aggregates (Figure 30). No mitotic cells were found expressing the construct.
-N vs C- GFP expression: Not confirmed.

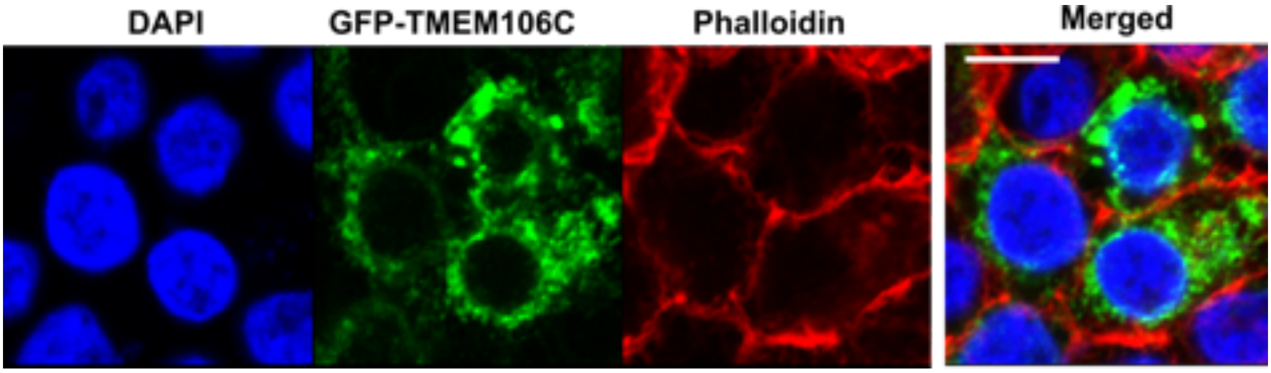

Figure 30. GFP-TMEM106C expression. Interph ase: GFP signal localised on nuclear membrane and cytoplasmic aggregates.

Gene Symbol: ***ZIK1***

Gene description: zinc finger protein interacting with K protein 1
ZIK1-GFP: No GFP signal.
GFP-ZIK: Interphase: GFP signal concentrated in nuclear bodies (Figure 31).
-N vs C- GFP expression: Not confirmed.

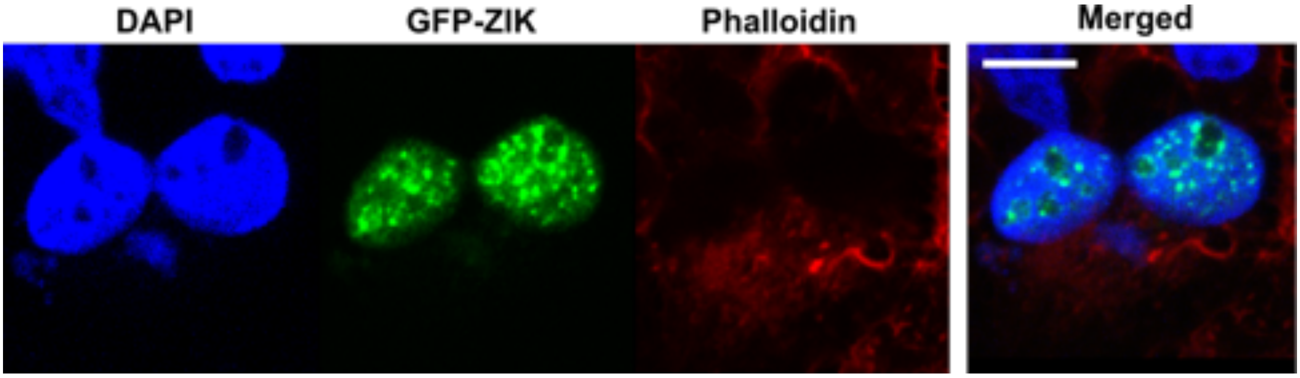

Figure 31. GFP-ZIK expression. Interphase: GFP signal concentrated in nuclear bodies.
