# Supplementary material for: Assembly of a parts list of the human mitotic cell cycle machinery
Source: J Mol Cell Biol. 2018 Nov 17;11(8):703–18. doi: 10.1093/jmcb/mjy063 (PMC6788831; doi:10.1093/jmcb/mjy063)
Supplement: mjy063_Supplementary_Data_S2 [file mjy063_supplementary_data_s2.pdf]

Supplementary Data 2. Results of RNAi screens of on cell cycle proteins.

In order to provide evidence that these unknown cell cycle-associated genes are involved in cell proliferation, we developed an esiRNA screening assay to identify the effect of gene knock down on the cell proliferation by using a RTCA system.

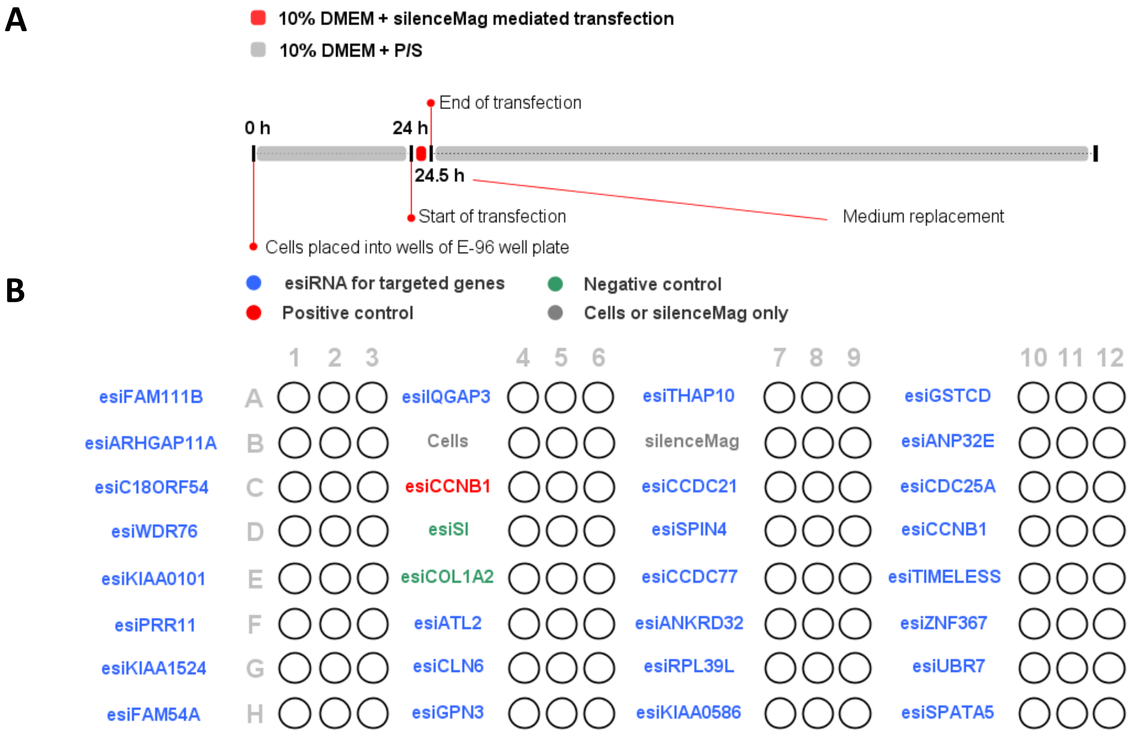

Figure 1 Experimental design of 96 well E-plate for real time RNAi screening. A) Time line of experiment from cell plating at t = 0 h to transfection conducted by silenceMag^{TM} at 24 h for 30 min followed by replacement of medium. The development of RNAi on cell proliferation was then monitored by RTCA system. **B)** Example of the layout of cells in a 96 well E-plate given esiRNA targeting the genes of interest.

Set up

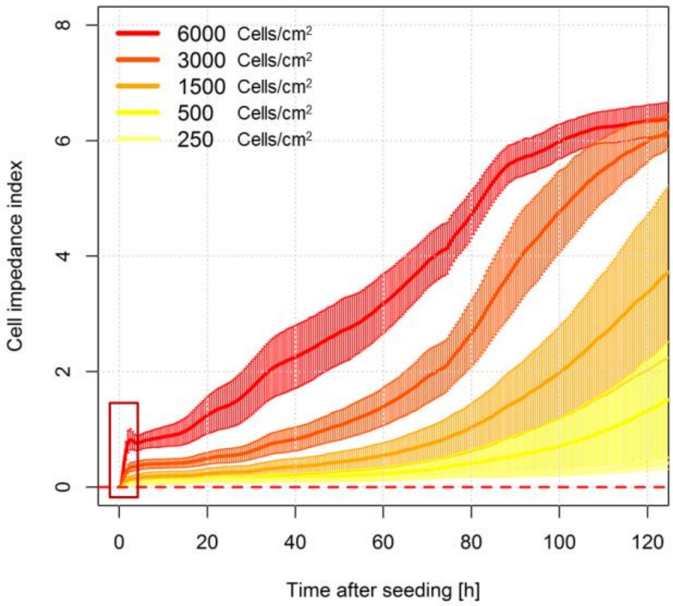

Figure 1. Duration of exponential phase was positively correlated with serially diluted seeding densities. The length of exponential phase in cell proliferation was positively correlated with serially diluted seeding densities of NHDF cells at 250, 500, 750, 1,500, 3,000, and 6,000 cells/cm^2 in E-plate 96. The CI over time of serially diluted seeding densities was profiled by RTCA.

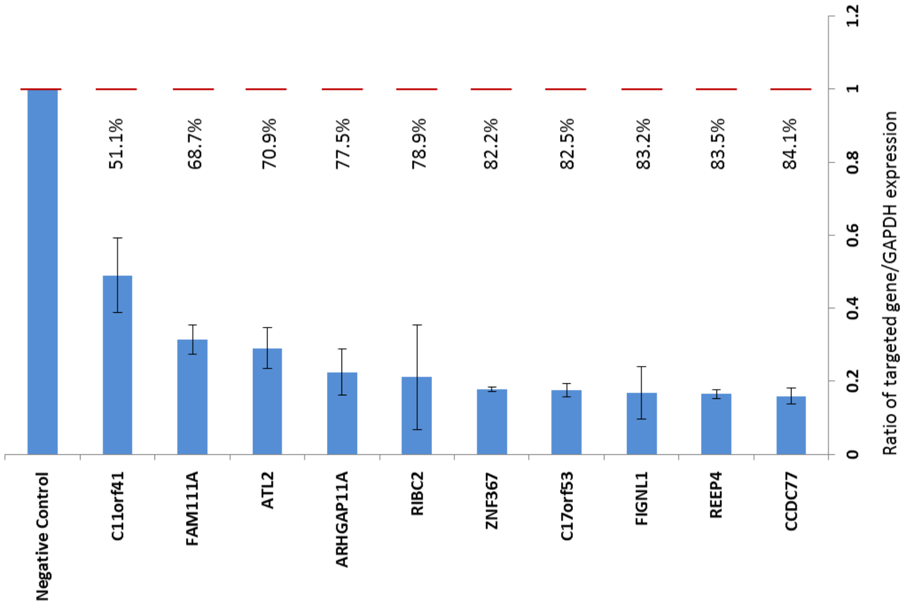

Figure 3. Knock down efficiency of genes identified as activators in in dividing cells. % of knock down efficiency was calculated between the expression level of targeted genes and housekeeping gene *GAPDH*. Data shown as mean ± SD of replicates (n = 3). Expression ratio was normalized relative to the housekeeping control (negative control), GAPDH.

Growth profile of putative cell cycle associated genes with effect

A

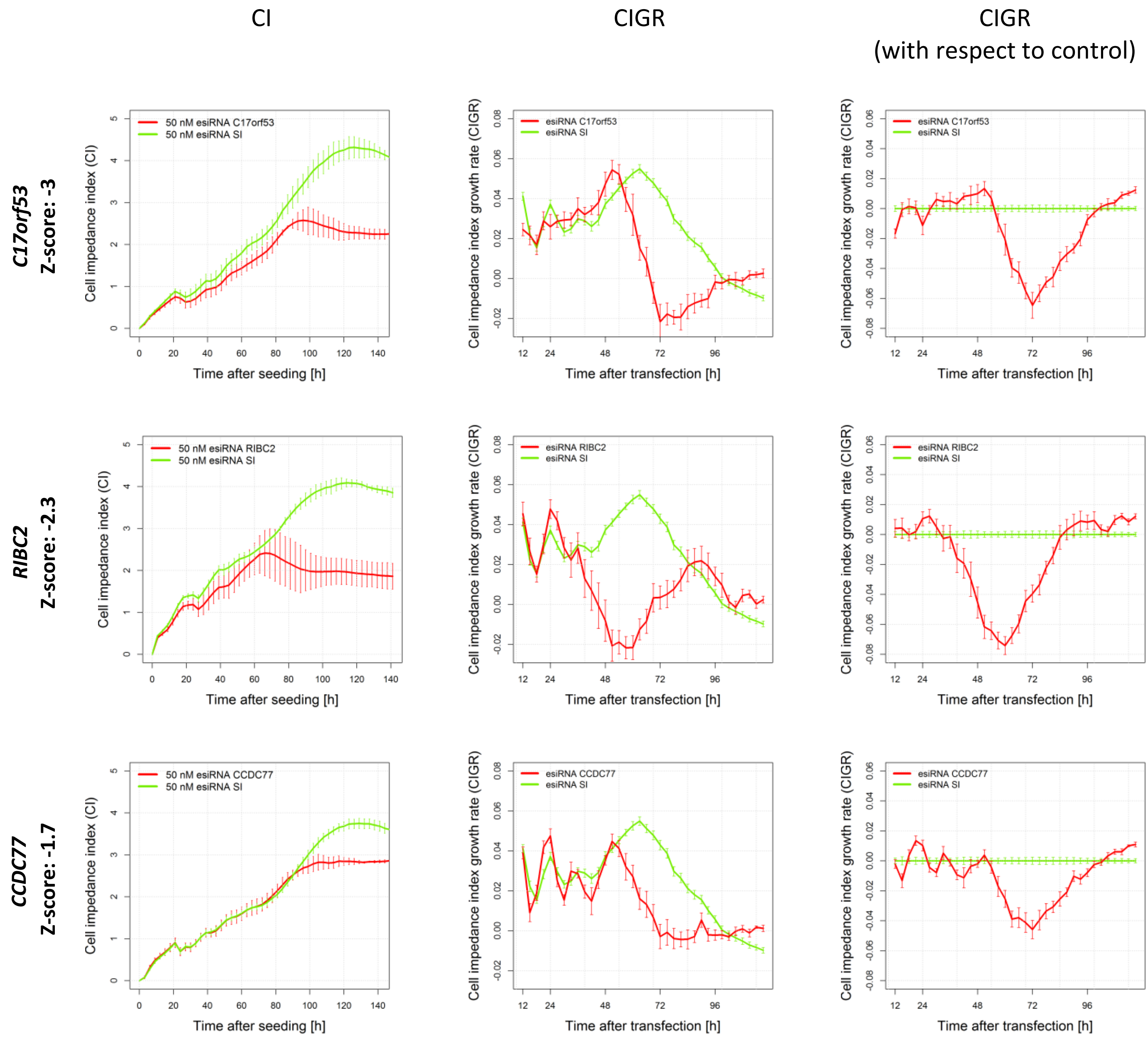

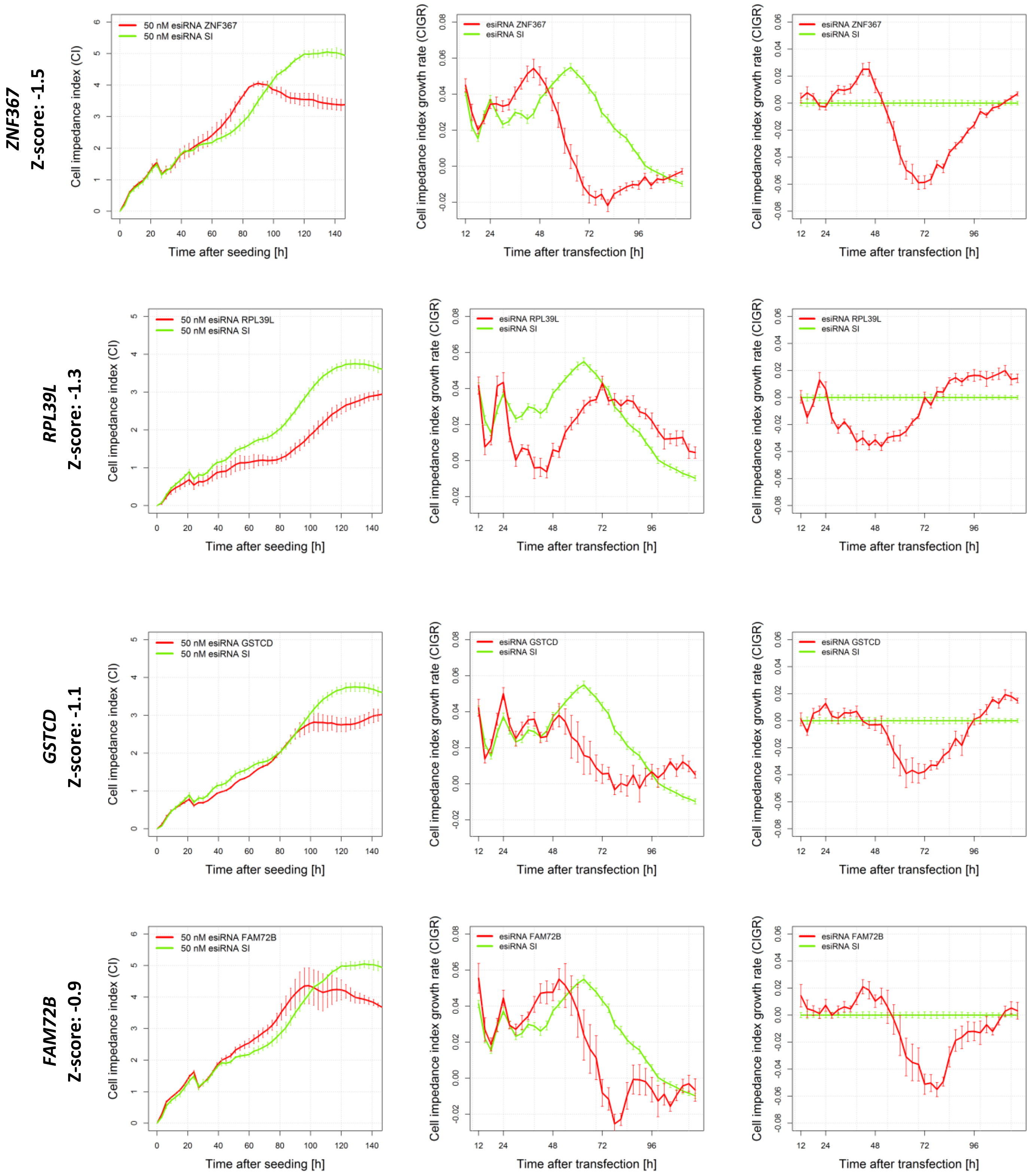
ZNF367
Z-score: -1.5
50 nM esiRNA ZNF367
50 nM esiRNA SI
Cell impedance index (CI)
Time after seeding [h]
esiRNA ZNF367
esiRNA SI
Cell impedance index growth rate (CIGR)
Time after transfection [h]
RPL39L
Z-score: -1.3
50 nM esiRNA RPL39L
50 nM esiRNA SI
esiRNA RPL39L
esiRNA SI
GSTCD
Z-score: -1.1
50 nM esiRNA GSTCD
50 nM esiRNA SI
esiRNA GSTCD
esiRNA SI
FAM72B
Z-score: -0.9
50 nM esiRNA FAM72B
50 nM esiRNA SI
esiRNA FAM72B
esiRNA SI

NEMP1
Z-score: -0.9

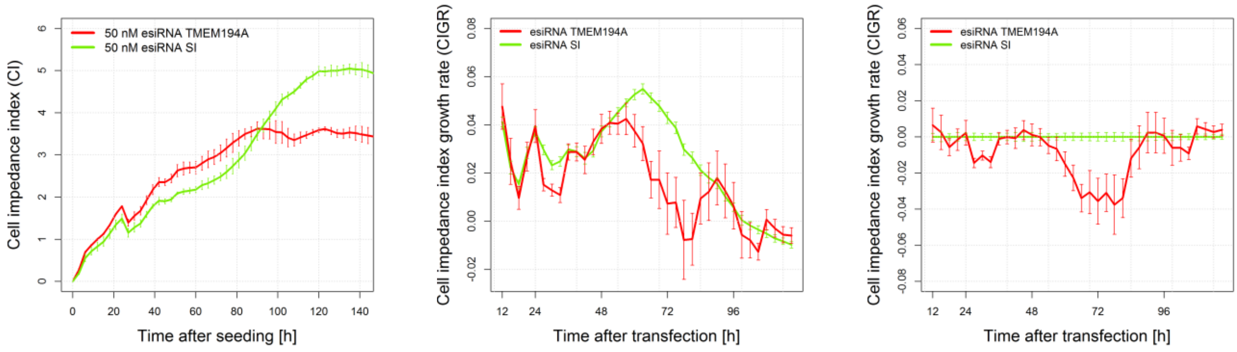

UBR7
Z-score: -0.9

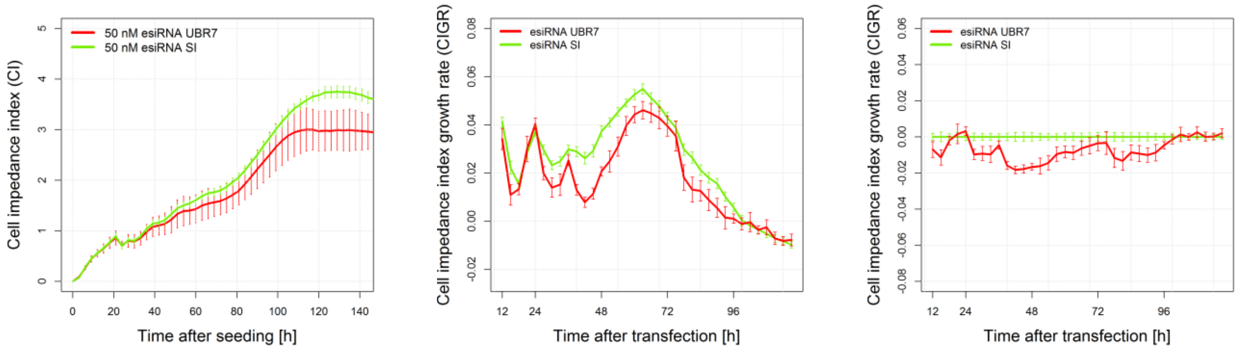

DEPDC1B
Z-score: -0.5

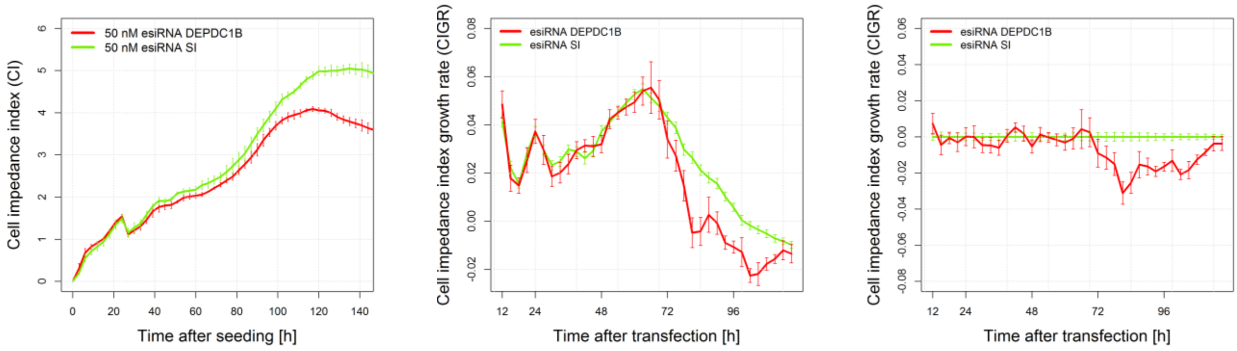

Known cell cycle-associated genes with effect on proliferation

B

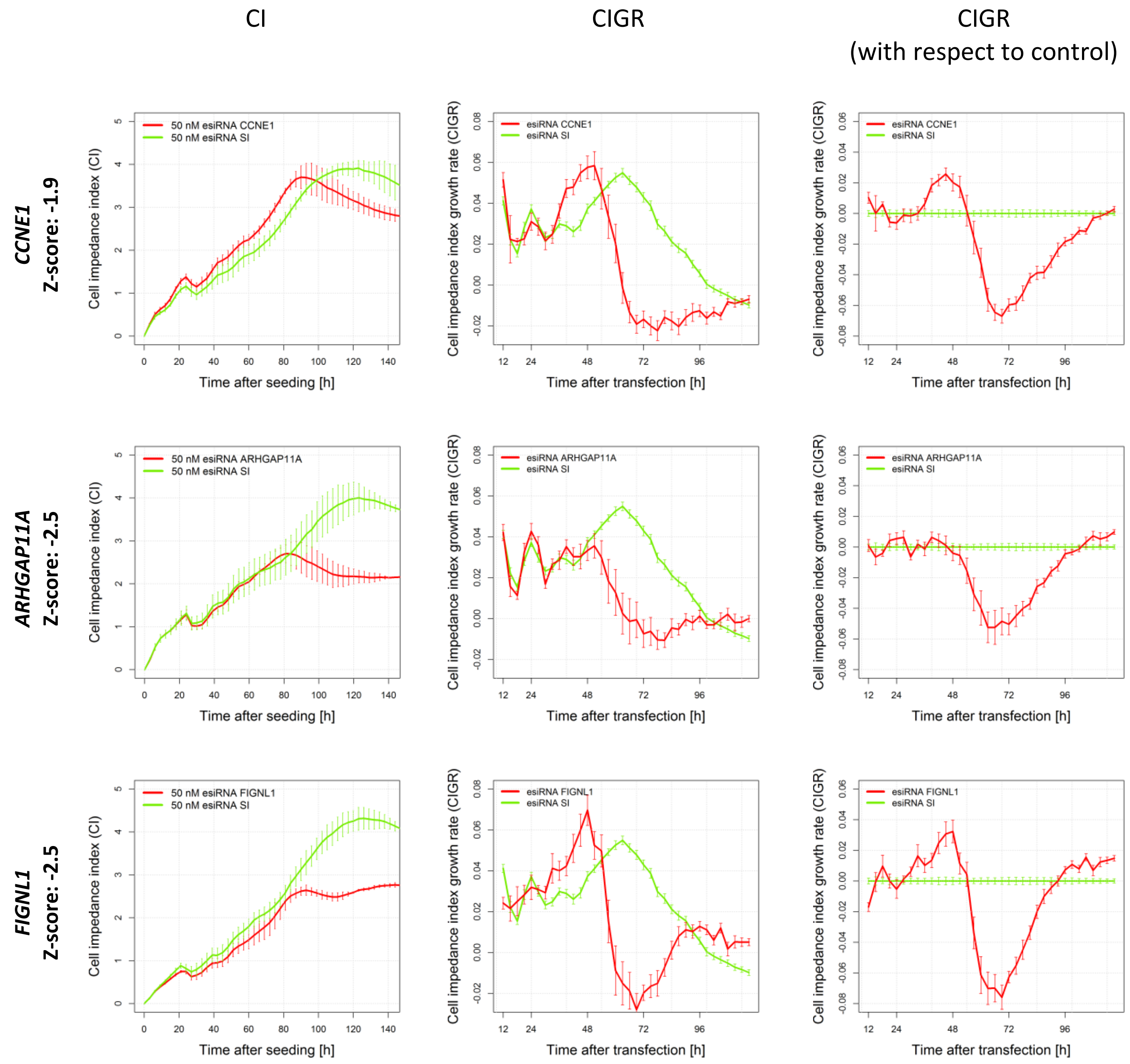

FAM111A Z-score: -2.4

CEP85 Z-score: -0.8

CENPA Z-score: -0.6

GINS1 Z-score: -0.2

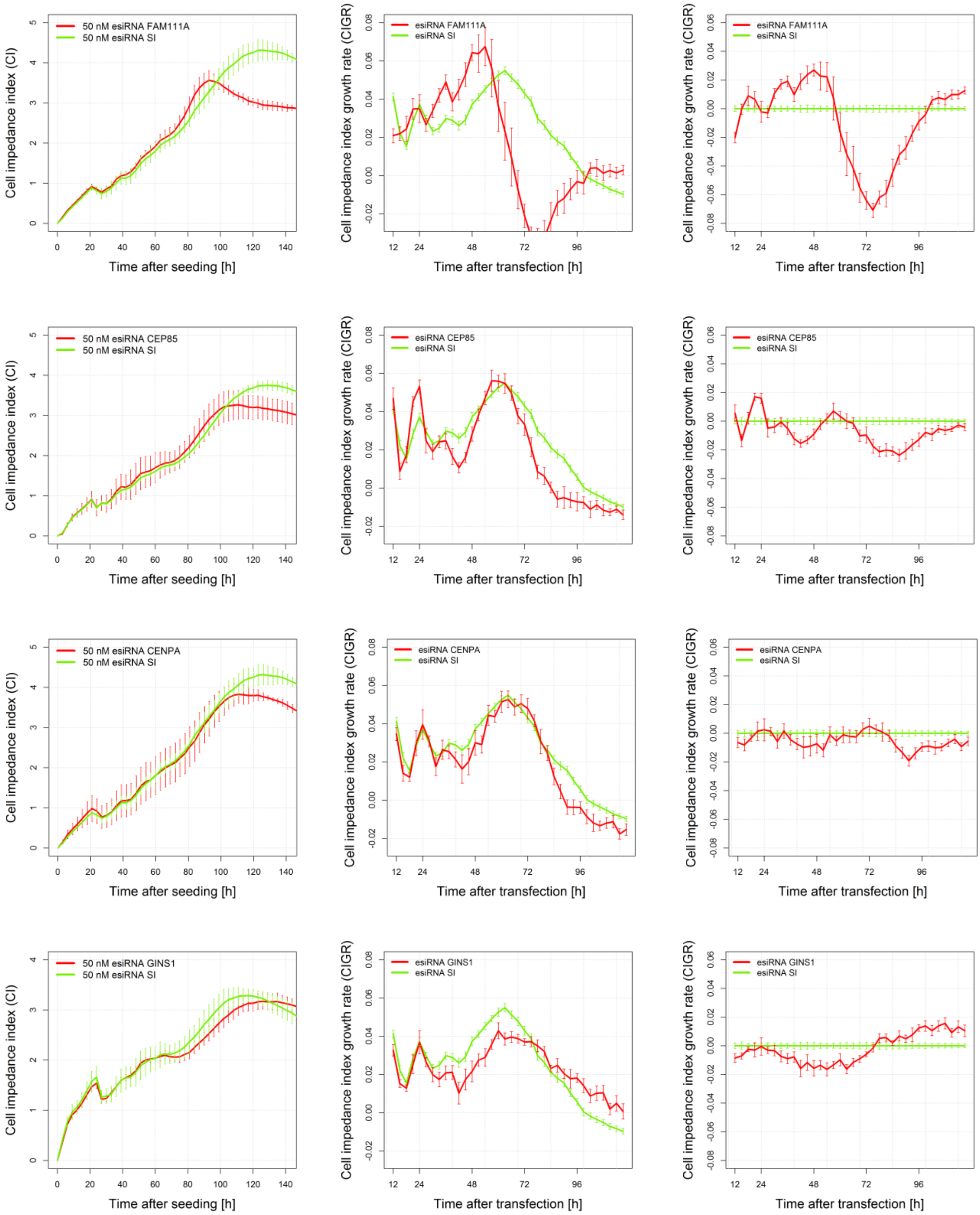

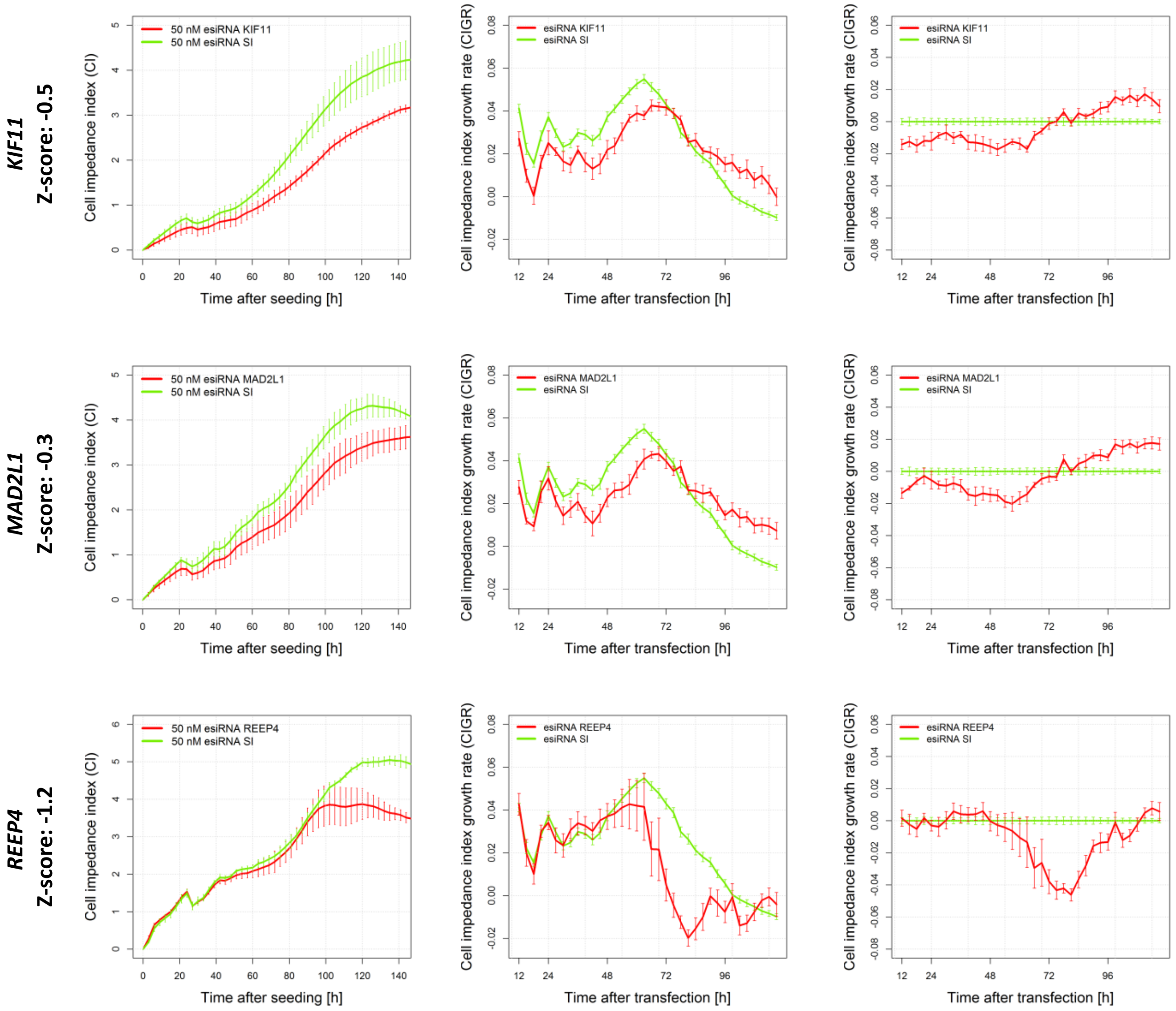

Figure 3 RTCA profiles of the trace of CI, CIGR, and CIGR with respect to time. The trace of CI for each gene in RNAi screening is composed of technical triplicates in each 96 well E-plate and E-plate were repeated three times. Plots of RTCA profiles were demonstrated in CI, CIGR, and CIGR with respect to negative control over time. For the column of CI over time, the transfection is at 24 h after cell plating. Red line indirectly represents that cell proliferation before and after transfection. Green line represents the negative control. Error bars represent mean ± SD. For the trace of CIGR and CIGR with respect to control over time, red line represents the CIGR of cells after transfection. Green line represents the negative control. Error bars represent mean ± SE. RTCA profile of tested genes were categorized based on the Z-score cut-off in **A)** Putative cell cycle-associated genes potentially essential in cell proliferation, **B)** cell cycle-associated genes known to be essential in cell proliferation.
