# Supplementary material for: Assembly of a parts list of the human mitotic cell cycle machinery
Source: J Mol Cell Biol. 2018 Nov 17;11(8):703–18. doi: 10.1093/jmcb/mjy063 (PMC6788831; doi:10.1093/jmcb/mjy063)
Supplement: mjy063_Supplementary_Figures [file mjy063_supplementary_figures.pdf]

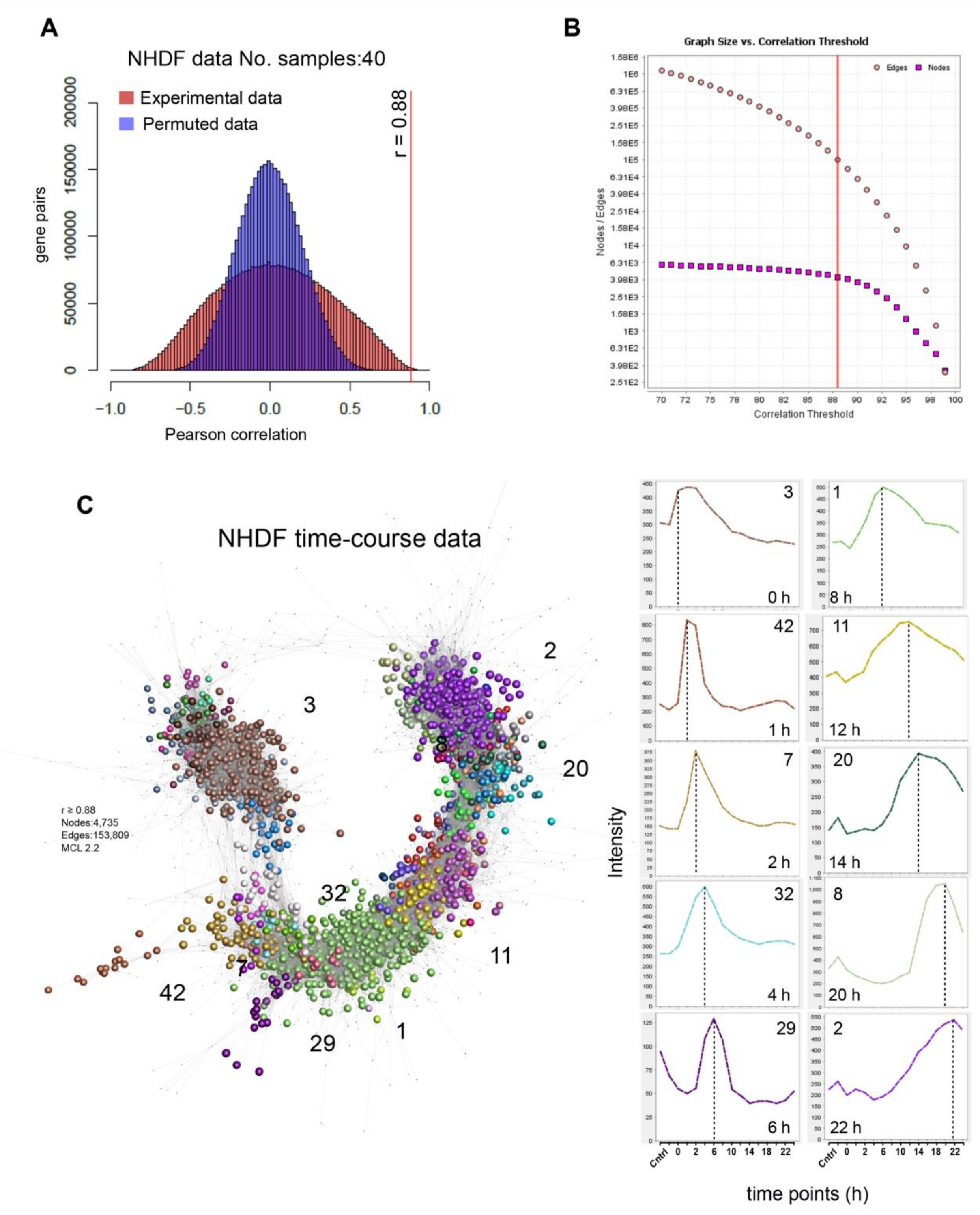

Supplementary Figure S1. Related to Figure 1. Analysis of NHDF transcriptomics data. (A) Plot shows the distribution of correlation values between 2,000 genes randomly selected from the NHDF data compared with that of the same genes but with permuted values. The threshold used for analysis excludes random correlations. **(B)** Plot showing number of edges and nodes as a function of the

correlation coefficient. A threshold of $r \geq 0.88$ was selected to include a minimal number of edges, while retaining a large number of nodes. **(C)** Network graph of the data clustered at MCLi 2.2 and average expression profiles of the main clusters showing gene expression as a function of time.

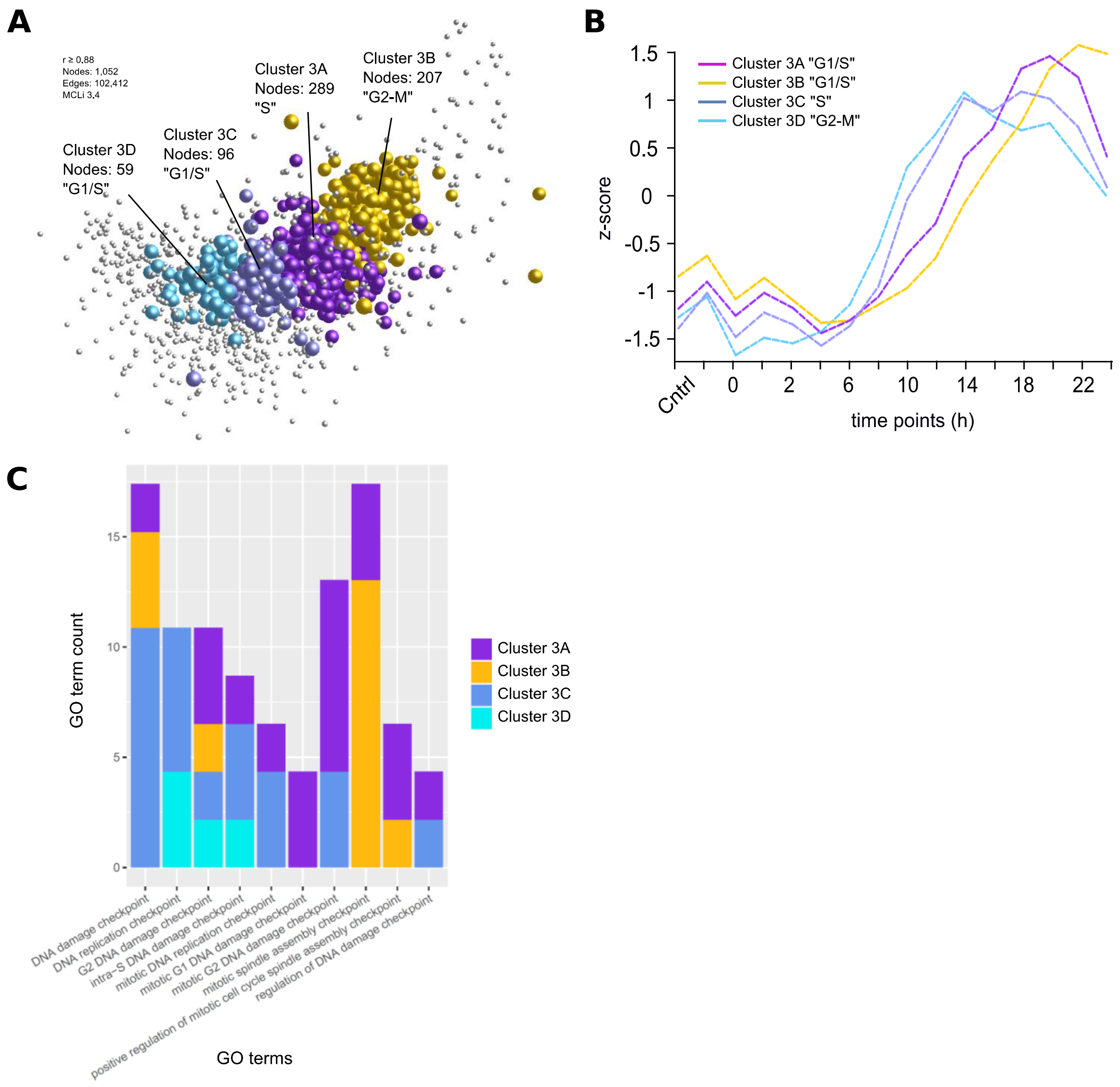

Supplementary Figure S2. Related to Figure 1. Sub-clustering of the NHDF_C3 cluster. (A) Network graph of the NHDF_C3 cluster re-clustered with higher cluster granularity (MCLi 3.4). Within the sub-clusters generated, four clusters were most representative of the core cell cycle phases 'G1/S', 'S', and 'G2-M'. **(B)** Average expression profile of the four sub-clusters showing their ordered peak of expression. Expression profiles were transformed to z-score for clarity. **(C)** Stacked barplot showing the enrichment of checkpoint genes in the four clusters as reported by GO_BP.

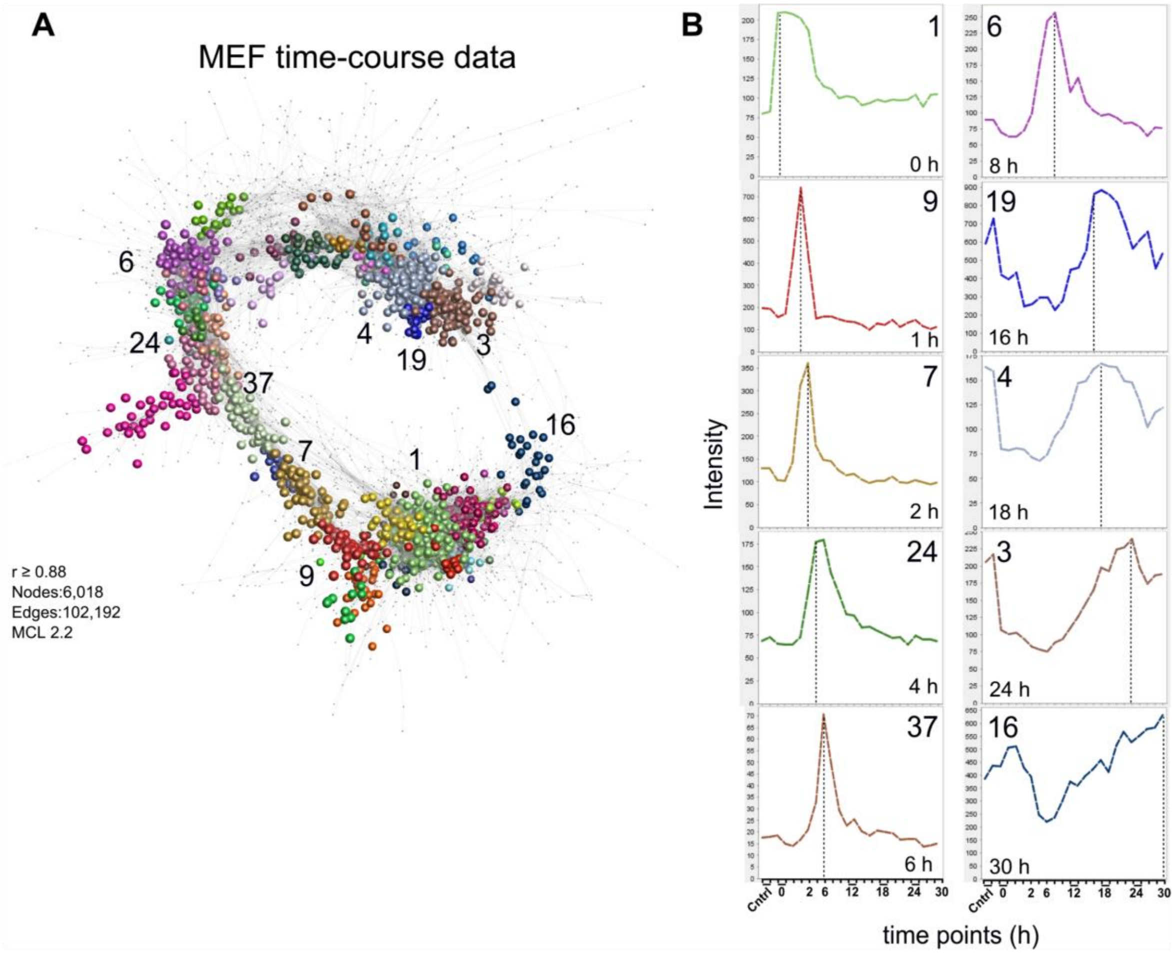

Supplementary Figure S3. Related to Figure 3. Network analysis of the MEF data. (A) Network graph of the MEF data (MCLi 2.2) at $r \geq 0.88$. **(B)** Average expression profile of the major clusters of cell cycle reulated genes.

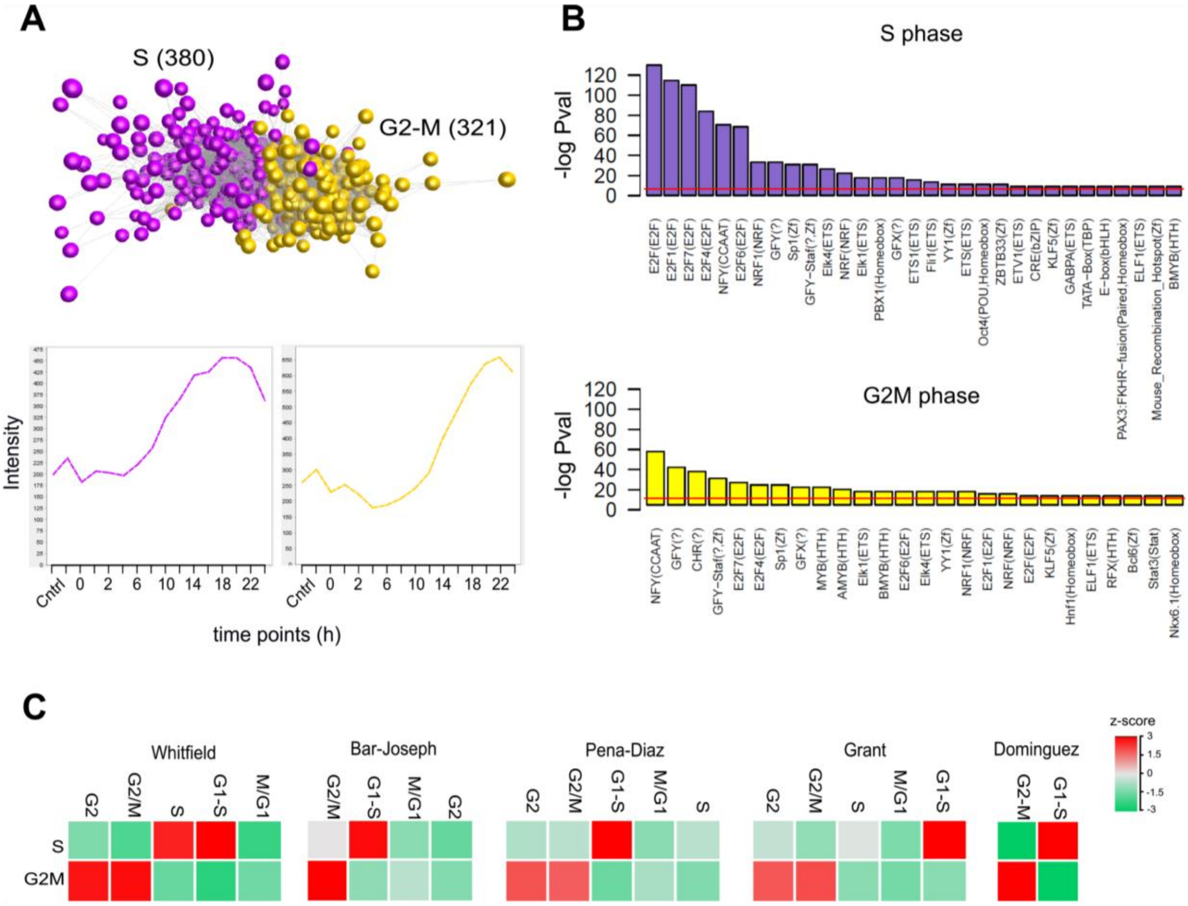

Supplementary Figure S4. Related to Figure 3. Phase assignation analysis and comparison with previous data. **(A)** The 701 genes associated with S/G2-M phase were assigned as being either 'S' or 'G2-M' phase according to their correlation with *bona fide* phase-specific cell cycle genes (see text), resulting in 380 S phase genes and 321 G2-M genes. **(B)** Motif enrichment analysis performed using HOMER were run on the two gene subsets returning significant enrichments for motifs bound by transcription factors known to be active in the corresponding phases. **(C)** Our phase assignation was compared to those of five previous studies. Heatmaps for each of these comparisons show overal consistent phase assignation.

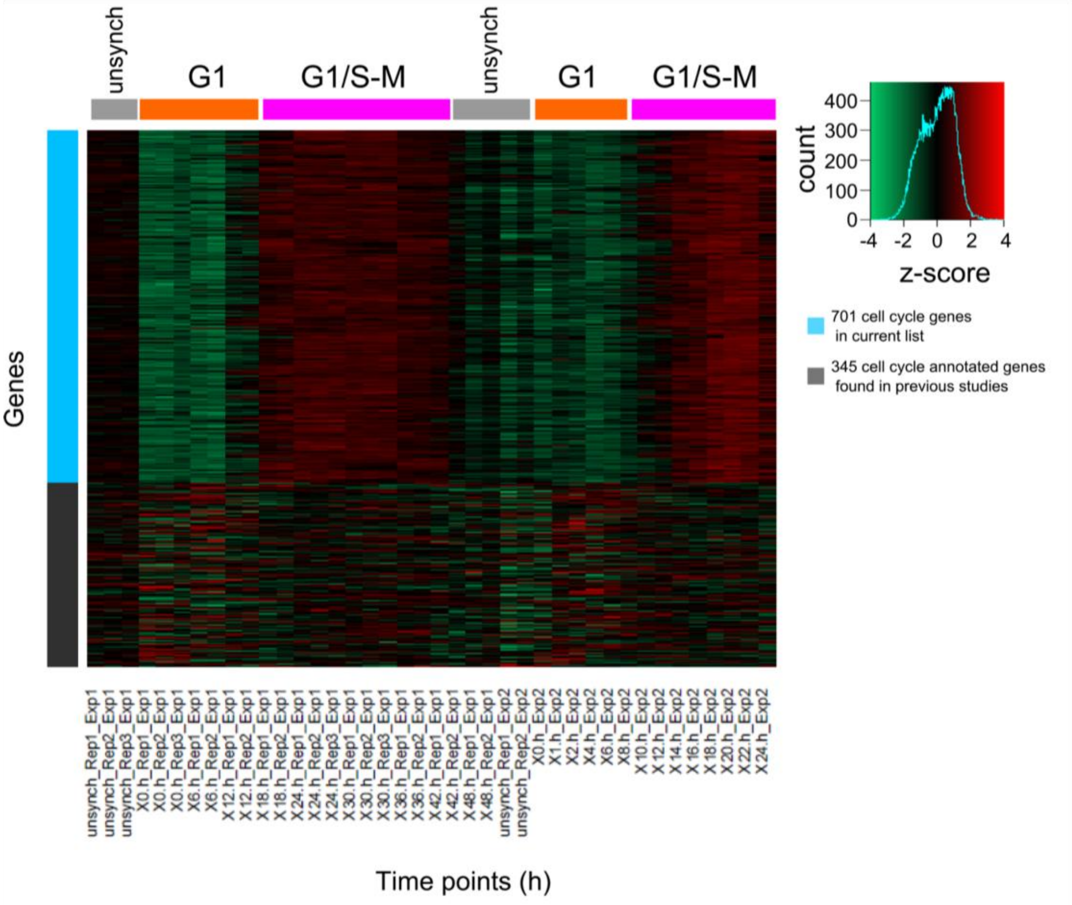

Supplementary Figure S5. Related to Figure 3. Expression of the 701 S/G2-M genes identified here and 345 other cell cycle-annotated genes. Expression of the 701 genes show up-regulation associated with entry into S phase throgh to the completion of M phase. The majority of the other 345 genes identified by the five previous cell cycle studies[13,14,18,34,35] but not in this study and associated with the GO_BP term cell cycle showed induction at earier time-points. Expression values were transfomed to z-score (see legend).

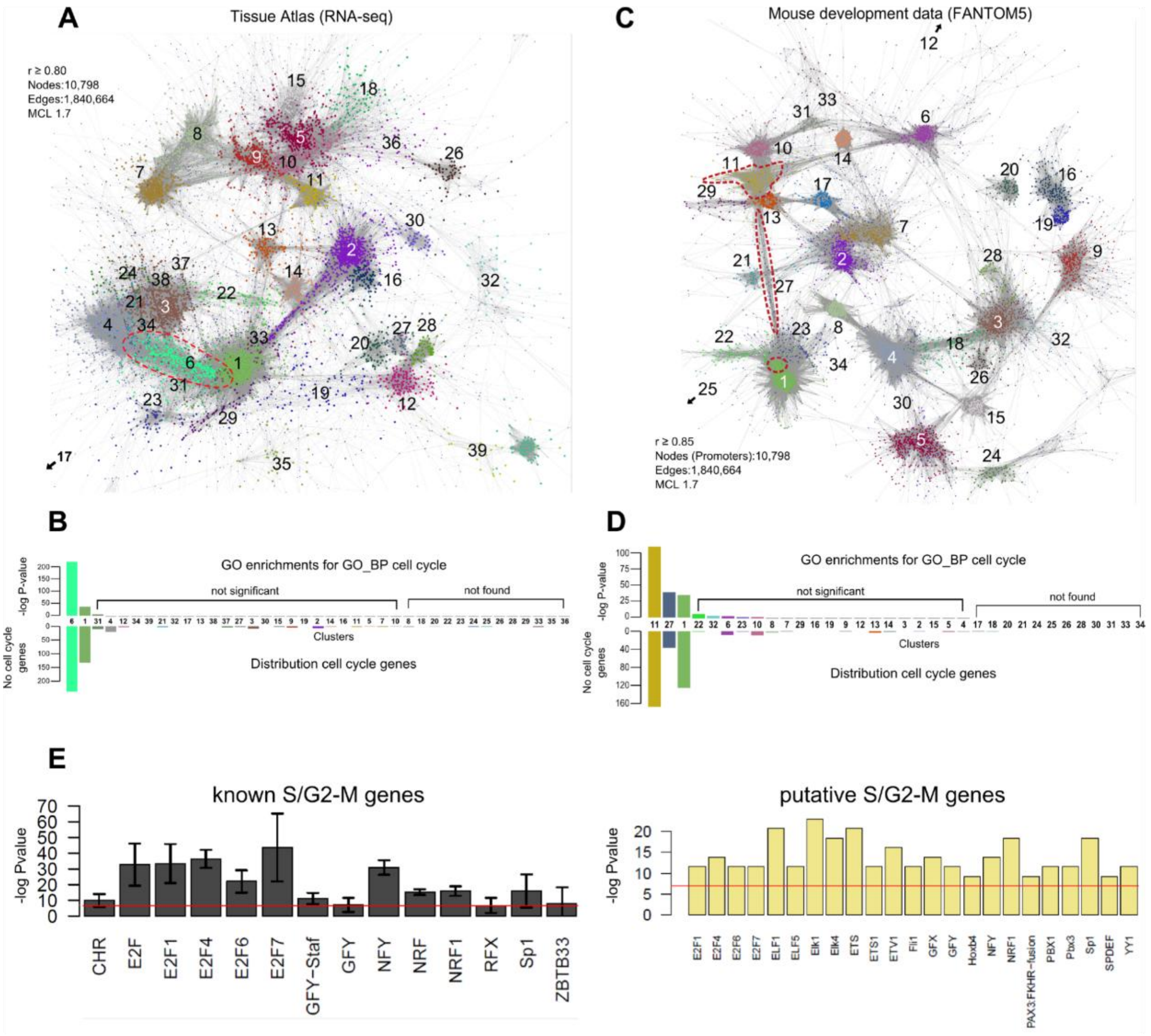

Supplementary Figure S6. Related to Figure 5. Coexpression networks of human tissue (HTA) and mouse tissue development (MDF5). (A) Clustered coexpression network of HTA analysis, showing clusters of genes exhibiting specific expression patterns. The location of the majority of cell cycle genes is highlighted by dotted red lines and in this graph reside in cluster HTA_C6. **(B)** Enrichment analysis shows this cluster to be highly enriched in genes with the GO_BP term cell cycle and with genes included in our list. **(C)** Clustered coexpression network of MDF5 analysis, showing clusters of genes exhibiting specific expression patterns. Cell cycle genes clusters are highlighted by dotted red lines. **(D)** Enrichment analysis shows clusters MDF5_C11, MDF5_C27 and MDF5_C1 to be significantly enriched with the GO_BP term *cell cycle* and with genes included in our list. **(E)** Motifs enrichments of ten randomly selected subsets of the known group of S/G2-M genes (left) equivalent to the number of putative S/G2-M genes (right). Significant cell cycle-associated TF, such as E2Fs and NFY, were identified in both cases.
